# Depuration Kinetics and Growth Dilution of Caribbean Ciguatoxin in the Omnivore *Lagodon rhomboides:* Implications for Trophic Transfer and Ciguatera Risk

**DOI:** 10.3390/toxins13110774

**Published:** 2021-11-01

**Authors:** Clayton T. Bennett, Alison Robertson

**Affiliations:** 1 School of Marine and Environmental Sciences, University of South Alabama, Mobile, AL 36688, USA; claybennett08@gmail.com; 2 Dauphin Island Sea Lab, Dauphin Island, AL 36528, USA

**Keywords:** *Lagodon rhomboides*, pinfish, bioaccumulation, depuration, ciguatoxin, Caribbean ciguatoxin, ciguatera, growth dilution, model, kinetics

## Abstract

Modeling ciguatoxin (CTX) trophic transfer in marine food webs has significant implications for the management of ciguatera poisoning, a circumtropical disease caused by human consumption of CTX-contaminated seafood. Current models associated with CP risk rely on modeling abundance/presence of CTX-producing epi-benthic dinoflagellates, e.g., *Gambierdiscus* spp., and are based on studies showing that toxin production is site specific and occurs in pulses driven by environmental factors. However, food web models are not yet developed and require parameterizing the CTX exposure cascade in fish which has been traditionally approached through top-down assessment of CTX loads in wild-caught fish. The primary goal of this study was to provide critical knowledge on the kinetics of C-CTX-1 bioaccumulation and depuration in the marine omnivore *Lagodon rhomboides*. We performed a two-phase, 17 week CTX feeding trial in *L. rhomboides* where fish were given either a formulated C-CTX-1 (*n* = 40) or control feed (*n* = 37) for 20 days, and then switched to a non-toxic diet for up to 14 weeks. Fish were randomly sampled through time with whole muscle, liver, and other pooled viscera dissected for toxin analysis by a sodium channel-dependent MTT-based mouse neuroblastoma (N2a) assay. The CTX levels measured in all tissues increased with time during the exposure period (days 1 to 20), but a decrease in CTX-specific toxicity with depuration time only occurred in viscera extracts. By the end of the depuration, muscle, liver, and viscera samples had mean toxin concentrations of 189%, 128%, and 42%, respectively, compared to fish sampled at the start of the depuration phase. However, a one-compartment model analysis of combined tissues showed total concentration declined to 56%, resulting in an approximate half-life of 97 d (R^2^ = 0.43). Further, applying growth dilution correction models to the overall concentration found that growth was a major factor reducing C-CTX concentrations, and that the body burden was largely unchanged, causing pseudo-elimination and a half-life of 143–148 days (R^2^ = 0.36). These data have important implications for food web CTX models and management of ciguatera poisoning in endemic regions where the frequency of environmental algal toxin pulses may be greater than the growth-corrected half-life of C-CTX in intermediate-trophic-level fish with high site fidelity.

## 1. Introduction

Benthic dinoflagellates have the capacity to produce a diverse suite of bioactive secondary metabolites that have been linked with seafood safety and human health concerns globally. One such group includes the neurotoxic ciguatoxins (CTXs) that have been linked to ciguatera poisoning and have been associated with some species and strains of epi-benthic dinoflagellates from the genus *Gambierdiscus* and *Fukuyoa* [1,2,3,4,5]. Several studies have reported that the in situ dinoflagellate community assemblage can change with environmental factors (e.g., temperature) and that species and strains may have physiological niches [6,7,8]. Likewise, long-term field studies have demonstrated that the CTX load of field-collected benthic microalgae is: (1) asynchronous with *Gambierdiscus* abundance; (2) site specific; (3) seasonal; and (4) occurring in pulses in tropical reef ecosystems [6,9]. It has also been proposed that CTX environmental pulses are linked to the presence of highly toxic *Gambierdiscus* strains rather than high overall algal biomass [5,6,9,10] potentially reducing the effectiveness of genus-level monitoring for these benthic HABs in terms of risk reduction. This presents some questions on the subsequent CTX load and CTX pulses that may occur in other demersal marine biota feeding on potentially toxicogenic epiphytic algae, aquatic invertebrates (e.g., amphipods), and other small grazers.

While still a working hypothesis in the field, the bioaccumulated CTX load in fish collected from ciguatera poisoning hotspots (and therefore ciguatera risk) is a function of the rate of toxin production by epi-benthic dinoflagellates (conceptual model in Lewis et al. [11] and revisited in Lewis and Holmes [12]). However, there remains a gap in understanding the timing between these processes. Field-based studies have provided evidence of a temporal lag of months to years occurring between the environmental triggers that increase toxin production, toxicity in upper-trophic-level fish, and increasing ciguatera cases [13,14]. Further, high fish toxicity (and resulting ciguatera prevalence) has been observed in high-site-fidelity fish when toxigenic dinoflagellate abundance and toxicity are low (or absent), suggesting a shift towards a non-toxic area [15,16], which leads to an assumption that bioaccumulated CTX is persistent in fish after the dinoflagellate source declines. However, field studies lack the ability to properly investigate ecologically relevant exposure routes (e.g., dietary, respiration, dermal) or the rates of uptake and depuration once a CTX source is removed, because total source removal can only be assumed.

Early studies attempted to investigate the retention of CTX-like toxicity (as a proxy for CTX, which was yet to be structurally elucidated) in a laboratory setting using fish that had naturally incurred toxin. For example, in Hawaii, Takata and colleagues (reported by [17]) allowed wild-caught Lutjanids (*Lutjanus bohar*, *L. gibbus*) and a Serranid (*Variola louti*) to depurate in aquariums at various intervals up to 14 months, while Banner et al. [17] kept wild *L. bohar* in holding ponds up to 30 months. In both studies, fish at the end remained toxic when fed to cats and mongoose. Davin et al. [18] fed several species of marine and freshwater fish the ground flesh or extracts of barracuda (*Sphyraena*) or whole *Gambierdiscus* cells (both sourced near the Caribbean Antilles Islands), followed by depuration time up to 81 d. Many of the fish died or were intoxicated. Largemouth bass (*Micropterus salmoides*) fed a high dose of cells recovered behaviorally but remained moderately toxic by intraperitoneal injection to mice after 81 days of clean feed, but fish fed lower doses were non-toxic after depuration. Recently, more laboratory studies have focused on CTX elimination using several exposure methods and in a variety of organisms. For instance, Ledreux et al. [19] performed single-oral dose-recovery experiments with mullet (*Mugil cephalus*) fed *G. polynesiensis* cells and reported only 5% of the CTX activity occurred in tissues after 24 h when analyzed by the mouse neuroblastoma assay (N2a). Li et al. [20] described multiple tissue kinetics of three Pacific CTX congeners, namely P-CTX-1 (CTX1B), P-CTX-2 (52-epi-54-deoxyCTX1B), and P-CTX-3 (54 deoxyCTX1B) extracted from eel (*Lycodontis javanicus*). Toxins were added to a pelleted feed and given to juvenile orange-spotted grouper (*Epinephilus coioides*) for 30 d and subsequently depurated for 30 d. The authors reported that CTX declined exponentially in depuration in some tissues (including muscle); however, CTX burden by the end of the study was not significantly different from the levels measured prior to depuration [20]. In another recent report, Caribbean CTX (C-CTX-1) depuration was investigated in the muscle of the freshwater goldfish (*Carassius auratus*) following a 43 d daily feeding of naturally C-CTX incurred amberjack (*Seriola* sp.) flesh prepared in agarose [21]. Estimated CTX toxicity (concentration) in muscle was reported to decline by approximately 86% out to 60 d post-exposure, but other tissues were not analyzed. Most recently, a depuration experiment using juvenile lionfish (*Pterois volitans*) was reported after 30–41 d experimental feeding on the flesh of the naturally P-CTX-contaminated parrotfish (*Chlorurus microrhinos*) [22]. Extracts from pooled liver showed a gradual decline in CTX concentration in fish harvested during a 43 d depuration, where fish were switched to a non-toxic diet of farmed sea bream (*Sparus auratus*). These two latter studies report depuration in single tissues (muscle and liver, respectively) and provide informative data on trends in bioaccumulation and depuration following oral exposure but were unable to capture cross-tissue distribution and did not examine fish growth that may contribute to the change in CTX tissue concentrations, but not body burden, as suggested by Holmes et al. [23] in a recent field-based study and review. We propose that estimates of toxicokinetic rates that also incorporate growth dilution may be more valuable than CTX concentrations in tissues in studies aiming to predict or model CP risk in the food web across species.

In this study, we provide data on the kinetic rate of C-CTX-1 bioaccumulation and depuration in the ecologically relevant marine omnivore *Lagodon rhomboides* (pinfish). Replicate fish were either fed a C-CTX-1 (*n* = 40) formulated diet or matrix matched control (*n* = 37) for 20 d, and then placed into depuration for up to 99 d. CTX toxicity in the major tissue compartments of *L. rhomboides* were quantified and compared through time and across replicates compared to control fish using an in vitro mouse neuroblastoma (N2a-MTT) assay.

Sampling and CTX analysis of whole muscle, liver, and other pooled visceral contents (heart, spleen, gall bladder, intestine) throughout the time course revealed dynamic trends in tissue burden and kinetics. Further, modeling concentrations corrected for fish growth dilution revealed an increase in the estimated half-life of C-CTX-1 in *L. rhomboides*, highlighting growth as a major source of pseudo-elimination of accumulated CTX. This correction reveals that C-CTX (measured here as a CTX3C equivalents) can be retained for several months following removal from the toxin source and if not accounted for could result in error during CP risk assessment and analysis of food web transfer potential. This work should be considered when sampling mid-trophic-level fish with high site fidelity and in the development of temporal models of CTX cascades where the frequency of toxin pulses may be shorter than the CTX half-life in fish. These data have implications for CP risk analysis in fish and may help to explain high toxin loads in highly migratory species collected in non-endemic regions for ciguatera (e.g., barracuda, mackerel, amberjack).

## 2. Results

### 2.1. Experimental Design, Diet Formulation and Consistency

Two dual-phase experimental trials were conducted to assess the depuration of Caribbean CTX-1 (C-CTX-1) that had bioaccumulated in *Lagodon rhomboides* fed a control or low-dose CTX diet through time and were sampled according to Table 1. A laboratory formulated diet was prepared and analyzed for procedural consistency (Table 2). Parameters including water content, pellet weight, whole-food CTX concentration, and pellet CTX concentration were quantified in all three batches of CTX pellets and four batches of control food for each trial. Slight differences were recorded in the water content of pellets after drying; however, food preparation was identical and resulted in the equivalent pellet weights. See methods for full details on the preparation of the control and CTX formulated diet.

### 2.2. Fish Growth

The mean initial weight of fish in the first trial (24.4 ± 9.4 g) was smaller than the second (36.4 ± 5.9 g; *p* < 0.0001); however, feeding rate was normalized to each fish at the start of trials so that fish consumed proportionally similar food and CTX relative to their mass (Table 2). At initial, fish were fed approximately 1.8% by weight equaling an exposure rate of around 0.018 ng CTX3C eq. g^−1^ fish day^−1^ that declined to 0.015 ng CTX3C eq. g^−1^ fish day^−1^ due to fish growth by day 20 when CTX feeding was stopped. The growth rate constants (*k_g_*) of control and CTX-treated fish collected at the same time point were similar except on experimental day 20 in both trials (see Supplementary Table S1). This effect was resolved when CTX-fed fish were swapped to the non-toxic diet for depuration.

**Figure 1:**
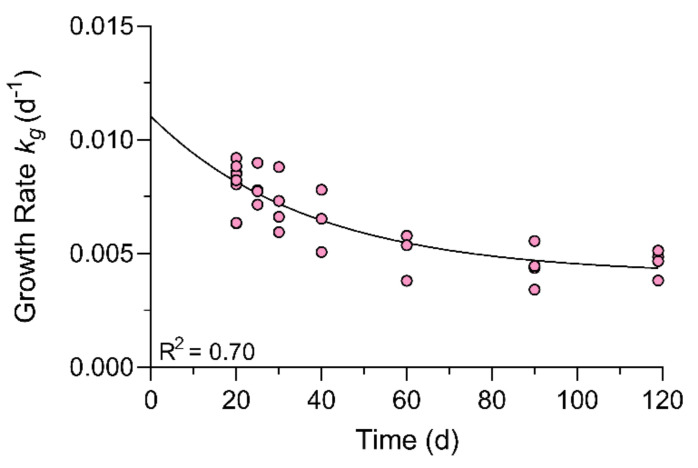
Non-linear regression of growth rate (*k_g_*) and time course in days of CTX fish in depuration.

Raw *k_g_* determined for the combined experiment between treatments were non-parametric according to statistical comparisons and visual inspection of the residual plots highlighted that the variability on day 6 and 10 d were responsible. Growth rates between replicate fish stabilized from day 10 to 20 (bioaccumulation) as fish stabilized to their control and experimental feeding regime. Exclusion of the data points in the early portion of the trial (day 1–10) restored normality and homoscedasticity. An ANOVA and post hoc analysis showed that the *k_g_* on days 20 and 25 were significantly different than days 90 and 119 in the CTX fish (Supplementary Figure S2B). The *k_g_* at day 40 was a transition period and was not significantly different than other time points. Non-linear regression using a one-phase exponential decay model to the *k_g_* of fish against time (R^2^ = 0.70) showed *k_g_* neared a plateau around experimental day 55 at approximately 4.07 × 10^−3^ day^−1^ (see Figure 1). The mean weight of CTX and control fish on the last day were 78 ± 13% and 65 ± 17% larger than at the beginning of the study which caused the daily feeding rate to decrease from around 1.8% to 1.0% of fish biomass by the end of the experiment.

### 2.3. Toxin Distribution in the Bioaccumulation Phase

Maximum concentrations of extracts used for CTX quantification were 50, 10, and 2.5 mg tissue equivalents (TE) well^−1^ (217.4, 43.5, and 10.9 mg TE mL^−1^) for muscle, liver, and viscera samples, respectively, and the mean value of the 75% maximal effective concentration (EC_75_) from CTX3C dose–response curves was 0.452 ± 0.151 pg well^−1^ (1.965 ± 0.657 pg mL^−1^). Based on these data the limit of quantification (LOQ) for each tissue was 0.009 ± 0.003 (muscle), 0.045 ± 0.015 (liver), and 0.181 ± 0.060 (viscera) ng CTX3C eq. g^−1^ tissue (Supplementary Figure S1). Brain was extracted and analyzed on a screening basis up to a 30 mg TE dose (130.4 mg TE mL^−1^) on the N2a-MTT, but all were non-toxic. Likewise, only 9 fish had gonads that were mature while all others were immature and unrecognizable. Mature gonads were extracted and assayed up to a 10 mg TE (43.5 mg TE mL^−1^) and were also non-toxic, therefore brain and gonads were excluded from further study. Quantifiable CTX3C eq. concentrations were possible in viscera across all sampling points, whereas the CTX toxicities in other tissues were detectable but not quantifiable (based on our QAQC criteria) until later time points.

For instance, CTX was detected on the first sampled time point (day 6) in all muscle extracts, but only one fish reached levels exceeding the LOQ. Likewise, CTX was not detected in 3 of the 4 fish livers from day 6 due to non-specific matrix effects on the N2a-MTT but were quantifiable from one replicate fish. After 10 d of CTX meals, 2 of 4 fish had CTX detectable in muscle and 2 of 4 fish sampled could be quantified. After 20 d of CTX feeding, all tissue extracts of sampled fish had toxicity above the determined LOQ and were the highest measured during the CTX feeding phase.

**Figure 2:**
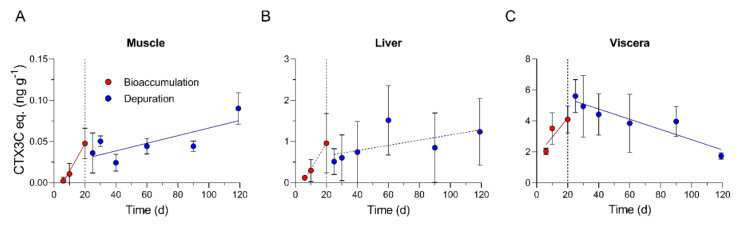
Linear regression of CTX-1 measured as a CTX3C eq. concentration (mean ± s.d.) in sampled (**A**) muscle, (**B**) liver, and (**C**) viscera through bioaccumulation (red) and depuration (blue). Solid regression lines denote a significant correlation while dashed lines were not significant due to high variability between individual fish within a tissue type. Control fish were negative for CTX in all cases and are not plotted. Data points represent the mean ± s.d. of replicate fish (*n* = 4 except on day 20 where *n* = 8 across two trials).

Mean CTX3C eq. concentrations in all tissues of fish harvested on day 20 were not different between trials based on a *t*-test (Table 3). Mean CTX3C eq. concentrations were significantly different in muscle on day 20 of trial 1 and trial 2 compared to day 6 (*p* < 0.05 and *p* < 0.001, respectively) and on day 20 (trial 2) compared to day 10 (*p* < 0.01; α = 0.05; Supplementary Figure S3A), but no statistical difference was found in liver or viscera of fish sampled during the bioaccumulation phase (day 6–20) based on ANOVA (Supplementary Figure S3B,C). A steady state of CTX concentration was not reached by day 20 based on the continued upward trend in CTX through the feeding period (Figure 2). Since this was not the goal of the present study, this was an acceptable outcome.

### 2.4. Toxin Distribution in the Depuration Phase

The primary goal of this study was to estimate depuration and based on the consistency of CTX3C eq. concentrations in fish analyzed at day 20 (see Table 3), we had confidence in combining sample data from the two identical trials for an extended depuration phase analysis (details in methods; summary in Table 1).

Mean CTX3C eq. concentration in muscle extracts were not significantly different between 0 and 70 d into the depuration period (Supplementary Figure S3A). The highest mean muscle CTX concentrations were measured in fish 99 d into depuration (day 119 of the complete study) and was 0.09 ng CTX3C eq. g^−1^ TE (Figure 2A). The mean concentration in muscle on the last day of depuration was significantly higher than those measured at all prior time points based on ANOVA, except for the day 20 fish from trial 2 which only marginally fell outside the criteria for significance (α = 0.05; *p* = 0.054; Supplementary Figure S3A). Consistent with these results were lower extract doses on the N2a-MTT needed for fish sampled on day 119 (5–20 mg TE) versus fish sampled earlier in the depuration phase (30–50 mg TE).

Liver extract CTX3C eq. concentrations from replicate fish in the depuration phase were the most variable of the tissues analyzed with relative standard deviation for trials 1 and 2 between 60 and 100% and 52 and 99%, respectively. This variability was consistent during both trials and through each sampling point with no significant trends (R^2^ = 0.09; *p* = 0.152; Figure 2B). Concentrations in liver of fish from trials 1 and 2 during depuration ranged from 0.12 to 1.77 and 0.23 to 2.52 ng CTX3C eq. g^−1^ TE, respectively, and no statistical differences were found between days 20 and 119 (α = 0.05; Supplementary Figure S3B).

Mean toxicity of viscera extracts were highest in fish sampled 5 d into the depuration phase (5.61 ± 1.06 ng CTX3C eq. g^−1^ TE; Figure 2C). No significant loss of CTX from viscera was measured by the end of trial 1 (day 20 = 4.40 ± 1.17 vs. day 40 = 4.43 ± 1.34 ng CTX3C eq. g^−1^ TE; Supplementary Figure S3C). When depuration time was extended in trial 2, a significantly lower concentration was measured on day 119 (1.73 ± 0.21 ng CTX3C eq. g^−1^ TE) than was measured 5 d and 10 d into depuration (day 25 vs. 119 *p* < 0.01; day 30 vs. 119 *p* = 0.018; Supplementary Figure S3C).

### 2.5. Muscle, Liver, and Viscera CTX Kinetics

Linear regression of the bioaccumulation phase data showed a positive correlation between the number of exposure days and CTX3C eq. concentration for muscle and viscera (R^2^ = 0.70, *p* = 0.0001; R^2^ = 0.47, *p* < 0.01, respectively; Figure 2A, and 2C; Table 4). The best fit models for bioaccumulation in both cases were simple straight-line equations (muscle and viscera AICc = 100 and 99.4%, respectively) in the form of y = *k_uptake_* ∗ x + b, where *k_uptake_* is uptake rate (ng CTX3C eq. g^−1^ fish day^−1^) equal to 3.37 × 10^−3^ for muscle and 0.124 for viscera (Table 4). Some liver samples on days 6 (*n* = 3) and 10 (*n* = 2) were excluded from the regression analyses due to significant non-specific matrix issues on the N2a-MTT assay because a value of zero could not be assumed. Liver concentrations during accumulation followed a non-significant increase (R^2^ = 0.24, *p* = 0.129; Table 4), so an uptake rate is not reported.

Straight line and one-phase exponential decay models were chosen for comparison of depuration kinetics of individual tissues. Depuration resulted in a linear decrease in viscera CTX3C eq. concentration (R^2^ = 0.44; *p* = 0.0005; Figure 2C), but an unexpected linear increase occurred with depuration time for muscle (R^2^ = 0.43; *p* = 0.0005; Figure 2A). The regression was likely skewed because of the weight of day 119 on the end of the regression line. Model comparison to describe depuration kinetics of tissues also showed simple linear models were the most probable in each case and depuration rates for muscle and viscera were −4.62 × 10^−4^ and 3.31 × 10^−2^, respectively (Table 4). However, liver CTX concentrations did not follow any pattern of depuration (R^2^ = 0.09; *p* = 0.152), so a depuration rate is not reported here.

### 2.6. Muscle, Liver, and Viscera CTX Burdens

Tissue burden, i.e., the amount of contaminant per tissue (ng CTX tissue^−1^) was calculated by multiplying the CTX concentration in each tissue by the whole tissue wet weight determined at the time of dissection. The concentrations of CTX (ng CTX g^−1^ TE) within *L. rhomboides* tissues were present at levels of descending concentration as follows: viscera > liver > muscle. However, the tissue CTX burden (that incorporates total tissue mass) showed some variability between individuals. For instance, viscera carried the highest toxin burden in all fish fed CTX throughout the entire experiment followed by muscle in 28 individuals. In the other 12 CTX-exposed individuals, liver CTX burden was slightly higher (1.4 ± 0.3 fold) than in muscle for 10 fish. CTX was not detected in muscle and liver of two fish at day 6 which prevented a comparison.

**Figure 3:**
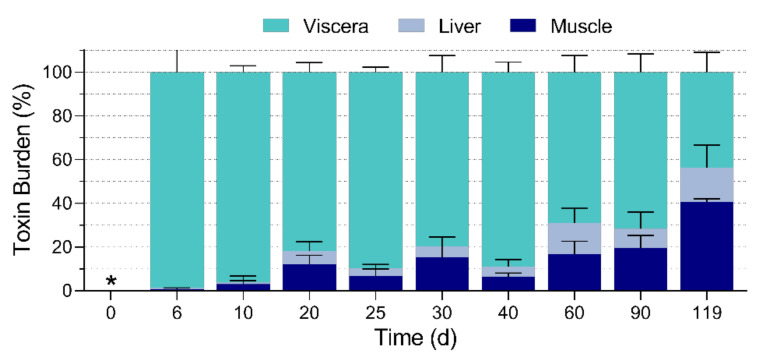
Distribution of the C-CTX-1 burden in tissues of *L. rhomboides* expressed as a percent of the whole burden measured in CTX3C eq. Note that fish were placed into depuration after day 20. Bars represent the mean ± s.d. of replicate fish (*n* = 4 except on day 20 where *n* = 8 across two trials). *CTX was below detectable levels on day 0.

Total CTX body burdens in fish were not directly compared across days because fish were fed different amounts of CTX based on individual starting body weight. Instead, toxin burdens of replicate fish were compared by the relative distribution of CTX as a percent of the sum of toxin burden measurements in tissue compartments (Figure 3). Up to day 40, viscera contained most of the total measured toxicity (89 ± 9%) followed by muscle (7 ± 4%) and liver (3 ± 2%). From day 40 to 119, the relative distribution of toxicity began to shift. While relative viscera burden declined to 44% the relative muscle burden increased to 41% on the last day.

### 2.7. One-Compartment Model Kinetics and Growth Correction

**Figure 4:**
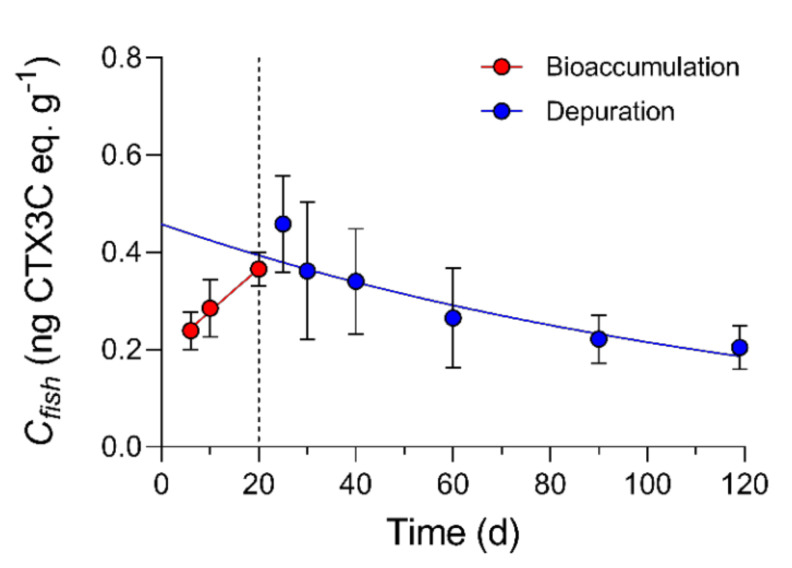
One compartment analysis of accumulated C-CTX-1 quantified by N2a-MTT as a PCTX3C eq. showed bioaccumulation (red) followed a linear pattern, while depuration (blue) followed exponential decay. The uptake rate (*k_uptake_*) calculated by linear regression was equal to 8.80 × 10^−3^ ng CTX3C eq. g^−1^ day^−1^ (R^2^ = 0.67, *p* = 0.0001) and logarithmic transformation of depuration data resulted in a first-order relationship (R^2^ = 0.43). The overall elimination rate constant (*k*_2_) was 7.161 × 10^−3^ day^−1^ based on the slope of the linear plot. Vertical dashed lines indicate the point where fish were swapped from CTX feed to a non-toxic food for the depuration phase. Data points represent the mean ± s.d. of replicate fish (*n* = 4 except on day 20 where *n* = 8 across two trials).

For the one-compartment model, the total concentration (*C_fish_*) represents the sum of CTX burdens in muscle, liver, and viscera divided by the combined mass of those tissues. The one-compartment model showed CTX bioaccumulation in *L. rhomboides* followed a linear increase from day 0 to 20 at an overall *k_uptake_* of 8.80 × 10^−3^ ng g^−1^ day^−1^ (R^2^ = 0.67, *p* = 0.0001; Figure 4).

**Figure 5:**
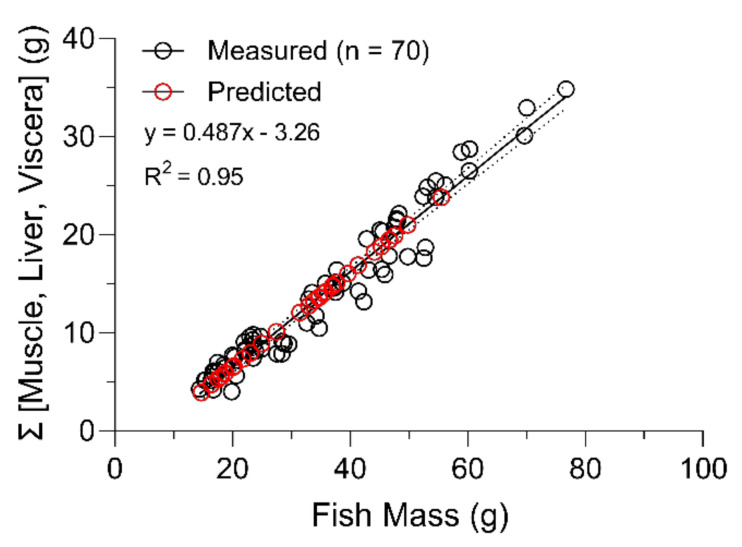
Combined sum of measured tissue masses (∑ [muscle + liver + viscera]) (black) of all *L. rhomboides* sampled in this study as a function of total fish mass. Total body masses at the start of depuration were estimated using an exponential growth formula (see Methods) and were used to estimate the ∑ [muscle + liver + viscera] (red) for each fish at experiment day 20 which entered the depuration phase (*n* = 24). Dashed lines are 95% confidence intervals.

**Figure 6:**
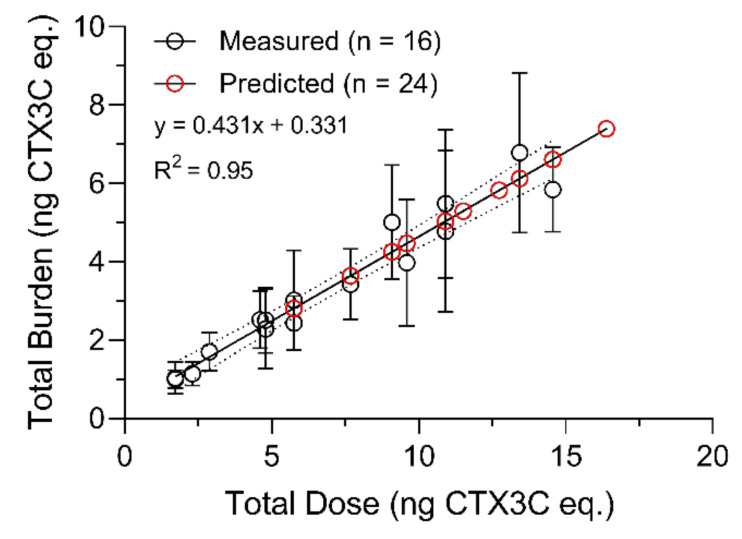
Linear correlation of cumulative CTX burden with total dose in *L. rhomboides* sampled during the bioaccumulation phase (black; *n* = 16; R^2^ = 0.95). The relationship was used to estimate the CTX burden at the start of the depuration phase (i.e., after day 20) for fish remaining after the bioaccumulation period that entered depuration (red; *n* = 24). Dashed lines are 95% confidence intervals. Error bars are standard deviation of the CTX burdens (measured concentration ± s.d. × tissue mass). Some points overlap.

The straight-line plot of the transformed total concentration data (Ln [*C_fish_*]) against time confirmed depuration approximately followed first-order kinetics (R^2^ = 0.43) and the overall *k*_2_ (linear slope) was equal to 7.161 × 10^−3^ with an estimated half-life of 97 d. Significant fish growth that was measured during the depuration phase meant the kinetic rate of elimination needed to be normalized to the CTX dilution rate due to increasing fish mass, a source of pseudo-elimination. Growth correction of *k*_2_ was investigated through multiple exponential decay simulations of the depuration phase data, where measured *k*_2_ is adjusted using measured fish growth rates (*k*_2 *−*_ *k_g_* = *k*_2 *growth-corrected*_) and based on estimates of initial CTX3C eq. concentrations (*C_fish(i)_*) immediately prior to depuration. The combined mass of whole muscle, liver, and viscera tissues (∑ [muscle + liver + viscera] g) had a strong linear correlation with whole body mass (R^2^ = 0.95; Figure 5) during the study, as did total accumulated burdens with cumulative doses at the end of the bioaccumulation phase (day 20) (R^2^ = 0.95; Figure 6) and provided a good estimate of initial CTX3C eq. concentrations (estimated *C_fish(i)_* = total CTX3C eq. burden/sum mass of whole muscle, liver, and viscera) in each fish that entered depuration.

**Figure 7:**
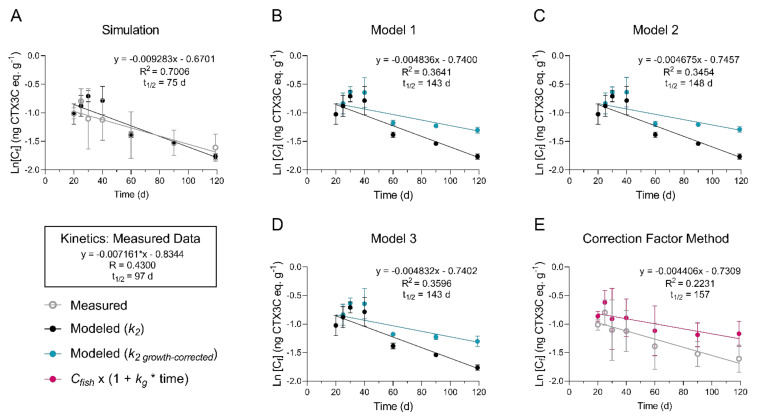
Modeled C-CTX-1 concentrations (based on CTX3C equiv.) compared to measured and growth-dilution-corrected models. Complete details on the (**A**) simulation model; (**B**) Model 1; (**C**) Model 2; (**D**) Model 3; are provided in the methods. (**E**) In the correction factor method, measured concentrations were adjusted using a simplistic approach where measured data were multiplied by a growth correction factor (i.e., *C_fish_* ∗ (1 + *k_g_* ∗ *t*_Δ *total*_)). Plotted symbols represent the mean ± s.d. (*n* = 4 except on day 20 where *n* = 8).

In the Simulation Model (Figure 7A), regression analysis with logarithmic transformation of the predicted *C_fish(t)_* values for each depuration time point (Ln [*C_fish(t)_*] *= -k_2_ ∗ t +* Ln [*C_fish(i)_*]) resulted in a predicted half-life of 75 d (R^2^ = 0.70; compared to 97 d from measured *C_fish(t)_*) and a correlation showed the Simulation Model fit closely to the measured *C_fish(t)_* (R^2^ = 0.68; *p* = 0.02). The Simulation Model was used as a starting point whereby several models were compared to assess the effect of growth. In Model 1, growth correction of the Simulation Model using the replicate average *k_g_* at each sampled time point (Figure 1; Table 5) increased the half-life to approximately 143 d based on model fitting parameters (Model 1: *k*_2 *growth-corrected*_ = 4.84 × 10^−3^; R^2^ = 0.364; Figure 7B). Growth correction for Model 2 was performed using three growth rates based on the ANOVA and Tukey’s test (Table 6; Supplementary Figure S1B) and produced a similar half-life at 148 d (Model 2: *k*_2 *growth-corrected*_ = 4.68 × 10^−3^; R^2^ = 0.345; Figure 7C). Likewise, growth correction using the *k_g_* measured for each individual fish resulted in a half-life of approximately 143 d (Model 3: *k*_2 *growth-corrected*_ = 4.83 × 10^−3^; R^2^ = 0.360; Figure 7D). Lastly, a common approach to growth correction was performed by multiplying the measured concentrations by a correction factor (1 + *k_g_* ∗ time). The correction factor method resulted in a half-life of approximately 157 d, although the uncertainty was greater in this model shown by the lower coefficient of determination (correction factor method: *k*_2 *growth-corrected*_ = 4.41 × 10^−3^; R^2^ = 0.22; Figure 7).

## 3. Discussion

This study is the first multi-tissue report on the dynamics of C-CTX elimination in an ecologically relevant intermediate consumer from the greater Caribbean region. This also represents the longest experimental C-CTX fish depuration in an important and rapidly expanding area of research. Since the initiation of these experiments in 2019, at least three similar experiments have been undertaken [20,21,22] and recently a conceptual model based on field-collected fish, focused on the contribution of growth in lowering CTXs was released [23]. Clearly, the knowledge gaps on CTX bioaccumulation and depuration kinetics have been acknowledged, and several groups are working to fill these. Modeling the disposition of CTX in wild reef fish is a next step for the development of predictive models on the time course of environmental changes, dinoflagellate proliferation and/ or toxification, and CP outbreaks.

### 3.1. Experimental Considerations and Outcomes

Pinfish (*L. rhomboides*) are omnivorous and undergo transitional feeding behavior during their life cycle. For instance, juvenile pinfish have a wide-ranging diet (small crustaceans, fish, polychaetes, tunicates, hydroids, seagrasses, epiphytes), while adults are reported to feed predominantly on epiphytes and macrophytes but continue to supplement their diet with many small invertebrates throughout their life [24,25,26,27]. As such, a generic trophic-level analysis can be difficult to determine based on the wide dietary range and multiple ontogenetic transitions during the lifecycle [25]. Pinfish are found from the coasts of New England to Brazil, and are widely distributed throughout the Gulf of Mexico, Florida Keys, and Yucatan [28], where CP also occurs. Pinfish also represent a substantial portion of the biomass in seagrass meadows and other structurally complex habitats (e.g., mangrove propagules, reefs, docks) and are a major source of organic matter exported to reef fish during seasonal migrations towards offshore reefs (20–100 m deep) in the Florida Big Bend area [29,30]. The stomach contents of gag grouper, a commercially important species from offshore reefs, contained 47% pinfish during migration periods [31], highlighting this species as a key prey item for higher-trophic-level fishes that have been implicated in ciguatera. Pinfish have also been used to study brevetoxin accumulation and exposure of many other natural and human contaminants [32]. Wild-collected fish used in this study were collected from an area with low prevalence of CTX and in the absence of brevetoxin-producing *K. brevis* blooms; however, to ensure no naturally incurred levels of CTX or brevetoxin that may skew our results, fish were acclimated, and subsamples of the population were tested for CTX and brevetoxins immediately following collection and the beginning of our experimental trials to ensure no detectable levels.

Some of the differences in our experimental design and the design of prior studies were important for the study goals. There is a trade-off to consider for experiments where it is necessary to maintain equal treatment conditions including the exposure rate (feeding) across large groups of experimental replicates. Since space was limited, alternatives such as grouping animals together in exposure tanks or pooling samples for analytical purposes, were considered, but risked the loss of statistical power and would introduce pseudo-replication. One of our goals were to capture the variability of CTX uptake and depuration in this species, so while fish had some minor size variability at the start of the trials, feed rations based on individual fish mass were prepared and maintained to ensure a consistent daily intake. Now that we have characterized the variability in these fish, we may be able to reconsider these alternatives in future studies to include changes in CTX metabolite formation and distribution via LC-MS/MS strategies (that require greater biomass for the CTX doses used here). The commercial omnivorous fish food that was mixed to create the pellets provided supplemental nutrition and acted as a binding agent which prevented fragmentation and loss of the powderized barracuda during feeding. In other studies, control and treated diets were either not matrix matched [33] or controls were not used [19]. In this case we were able to track similar growth rates between control and treated fish across trials since both of these aspects were controlled. The regulated feeding rate also led to a strong correlation (day 0–20; R^2^ = 0.95) between cumulative burdens and the ingested dose in fish sampled during the CTX feeding phase as reported by others [33]. This correlation was used to estimate the accumulated CTX burden in fish prior to their depuration and allowed the analysis of the change in total body concentration of individuals over time under multiple projections using growth correction which was also attempted in one prior experimental study [20]. The isolation of fish, controlled feeding rate, and sufficient replication also are important to capture the individual physiological variability in CTX toxicokinetics, but these have not always been met in other CTX studies [21]. Parallel controls at each sampling time point (except experimental day 90) provided both growth and behavioral assessment (no behavioral issues observed) and analytical controls for the tissue analyses by the N2a-MTT assay.

The procedural consistency across trials was crucial for us to consider CTX distribution data through time as a single set in the analyses. Several points of data support our consistency in approach. Quality control data shows reproducible pellet toxicity and feeding rates across both trials which was supported by the cumulative burden measurements in fish related to the total dose (Figure 6). Further, four fish were analyzed in both trials on the last day of the bioaccumulation phase (day 20) and the toxicity of all tissues sampled were not significantly different (see Table 3). The controlled formulation of the experimental pellet diet also showed that pellet weight and proportional amount of food provided to each fish between control and treated groups was consistent. Based on the supporting evidence collected, we deemed it acceptable to combine datasets from the two experimental trials for analyses of the extended depuration kinetics.

Use of the N2a-MTT assay to determine tissue CTX concentrations has been widely accepted by the CP research community and seafood management agencies alike for several decades [34]. This methodology has been used in prior CTX exposure studies of various organisms to analyze CTX activity over time [19,21,35,36] and provides a measure of the composite toxicity of sodium channel activating metabolites that have implications for human health. In selecting N2a-MTT for our measurements we gained the sensitivity that we needed to evaluate toxicokinetics across individuals through time in this small species but lost the ability to evaluate trends in CTX profiles throughout the experiment. Unfortunately, we had insufficient tissue to support LC-MS/MS analyses without combining replicates (as in other studies) and we wanted to maintain and evaluate the variability between fish so that trends in depuration among tissues would be more robust. As LC-MS/MS methods for CTXs become more sensitive (or with the use of a larger species) we hope to revisit the change in toxin profiles (if any) through these processes in future work.

### 3.2. Muscle, Liver, and Viscera CTX Kinetics: Bioaccumulation

Prior to the study, fish were acclimated to the feeding schedule using cooked shrimp and transitioned to gel pellets (no barracuda added) over the 2-week period. By the start of experiments fish were consuming pellets immediately when added to the tanks and continued to do so throughout the entire study. Rapid consumption during the experiment likely limited leaching of toxin from the CTX-pellets into the aquarium water. Additionally, 35% water changes were performed weekly on all aquarium systems to maintain water quality and prevent accumulation of possible residual toxins in the tank water. Although tank water was not analyzed for CTX, the tanks of control and CTX-dosed fish shared the same recirculating artificial seawater by randomized design and none of the control fish tissue extracts or control feed exhibited CTX activity by the N2a-MTT. Additionally, tank substrate was regularly vacuumed by siphoning to prevent the re-uptake of any CTX in fish excrement. Therefore, we assumed with confidence that measured CTX activity was dominated via the dietary exposure route.

During the uptake phase (day 1–20), measured CTX concentrations in muscle and viscera of pinfish were positively correlated with number of CTX exposure days and followed linear trends (Figure 2). Toxin uptake rate estimated in pinfish muscle (0.003 ng CTX3C eq. g^−1^ TE day^−1^) by N2a-MTT was in the range of uptake rates reported for P-CTX-2 and -3 in juvenile grouper (*Epinephelus coioides*) measured by LC-MS/MS (0.001–0.005 ng g^−1^ day^−1^) but was 10-fold lower than P-CTX-1 (0.033 ng g^−1^ day^−1^) [20]. Toxin uptake rate in pinfish viscera (0.123 ng CTX3C eq. g^−1^ TE day^−1^) was not comparable to other studies due to sample preparation of this compartment; however, CTX bioaccumulation in viscera was >41 times the rate in pinfish muscle which led to extremely high visceral concentrations (4.40 ng CTX3C eq. g^−1^) versus muscle (0.04 ng CT3C eq. g^−1^) by the end of the bioaccumulation phase.

Between days 6 and 20, CTX concentrations in viscera were 42–330 fold (except 1 fish on day 6) and in liver, 3–69 fold higher than the concentration in muscle. The range of concentration ratios for liver to muscle are similar to what has been reported from fish collected in CP endemic waters [37]. However, as noted by Vernoux et al. [37], we did not detect any pattern in the concentration ratios (data not shown), and therefore support their recommendation to avoid using liver or viscera results to extrapolate flesh toxicity. These compartments may confirm CTX exposure in fish but would not reliably estimate risk of consuming fish fillets.

In some cases, the variability observed in CTX tissue concentration between individual fish replicates in this study was high. This variability was important to capture if we are to extrapolate future field studies where intraspecies variability will be even greater. The strength of the correlation between cumulative CTX burden and total ingested dose supports that fish were provided a normalized amount of CTX and nutritional food, as does our quality assurance/quality control data (Table 2 and Table 3). Our time until detection in the muscle and liver using the N2a-MTT assay was much longer than found for mullet, which occurred 3 hrs post-exposure to a single dose of *G. polynesiensis* cells [19] which could relate to species differences or the increased toxicity of P-CTXs produced by *G. polynesiensis* compared to C-CTX-1 that was used in this exposure study. Additionally, our CTX dose was approximately 15-fold lower (0.02 vs. 0.3 ng CTX3C eq. per g of body weight) to ensure that no behavioral disturbances were detected and supports an ecologically relevant range. Intraspecific variability in assimilating and compartmentalizing CTX also could have contributed to the limited detectability of CTXs in the muscle and liver during the first 10 days of the bioaccumulation phase. Omnivorous goldfish fed C-CTX-1 from *Seriola* sp. flesh at a rate of 0.014 ng CTX1B eq g^−1^ TE also had undetectable CTX in muscle after the first CTX dose but CTX was measurable (*n* = 2) by day 8 [21]. The dose and physiological differences between herbivorous and omnivorous fish may explain the observed lag time between the CTX activity of benthic dinoflagellates and herbivores compared to upper-trophic-level fish [6,13].

Through this work, we estimated an average net CTX assimilation of 43% in pinfish from the ingested dose throughout the bioaccumulation phase of this study (days 1–20; see Figure 6). The bioavailable amount of the CTX dose, which is the total CTX absorbed into systemic circulation, was not quantified here, but our estimate of net assimilation (bioavailable amount—first pass metabolism elimination) in pinfish was much higher than reported for C-CTX-1 in freshwater goldfish [21], P-CTX-1, -2, and -3 in juvenile grouper [20], and CTX3C in naso [33], which were all less than ~10%. In contrast, mullet assimilated 42% of ciguatoxicity from a single dose of *G. polynesiensis* [19] and orally bioavailable CTX in rats was calculated around 39% [35] which are congruent with measured levels in this study. The CTX burden in mullet declined to 5% of the single dose administered by 24 h, but repeated exposure was not evaluated [19]. It is also possible that C-CTX-1 investigated in this study has a higher assimilation compared to other CTX congeners, among other factors.

Considering the continued linearly increasing trend of CTX in each tissue during the exposure and bioaccumulation phase, we do not assume a steady state was reached during the CTX feeding phase. Clausing et al. [33] determined in herbivorous naso fed *G. polynesiensis* cells, that muscle CTX concentration stabilized somewhere between 8 and 16 weeks of exposure at bioaccumulated levels of approximately 3 ng CTX3C eq. g^−1^ TE. However, the CTX concentration in muscle of goldfish stabilized by day 29 at 0.03 ng CTX1B eq. g^−1^ TE [21]. This dissimilarity could be due in part to different physiology of freshwater and marine fish, but the possibility that the low sample size (*n* = 2) combined with biological variability prevented detectable trends in the goldfish study cannot be dismissed. Goldfish were reportedly not growing during the study whereas biomass of juvenile naso increased approximately four times compared with initial, therefore, the possibility that there may be differences in the steady-state concentrations reached between growing and non-growing fish cannot be ruled out.

### 3.3. Muscle, Liver, and Viscera CTX Kinetics: Depuration

Depuration resulted in an expected decrease in viscera CTX3C eq. concentration with time that followed a simple linear relationship (Figure 2C). Initially, however, the C-CTX-1 (measured as CTX3C eq.) concentrations in viscera continued increasing into the depuration phase and were at the highest measured levels five days after depuration was initiated (day 25 = 5.61 ng CTX3C eq. g^−1^). A similar trend was reported in a depuration study of juvenile grouper (*Epinephelus coioides*) where P-CTX- 1, -2, -3 concentrations in the skin and intestine were higher 4 days after depuration was started and then followed an exponential decline through the next 30 days [20]. One factor mentioned by the authors was that they observed some undigested material in stomachs when dissected, so this could have had an effect. In this study, we excluded stomachs to avoid possible crossover between assimilated and non-assimilated CTX. We did not measure fecal pellets or the tank water during the trials, so we were unable to capture the CTX levels eliminated from the organism directly; however, controls shared circulating tank water with CTX fish and no controls had detectable CTX levels supporting that dietary uptake was the only relevant exposure route.

Further explanation of the lag of CTX seen in the viscera compartment during this study could be attributed to the amount and quality of protein and the presence of carbohydrates and fiber in the pelleted food. Food quality has been well established as a factor that may increase gut retention and modify absorption kinetics for other toxicants in humans and mammals (see [38,39,40]), which could be examined in future studies for CTX. Variability of CTX kinetics, particularly from visceral organs, that has been reported between studies may be a function of dietary differences, in addition to species and experimental factors already described. Persistent viscera contamination could also be influenced by enterohepatic recirculation as seen for anthropogenic contaminants where a xenobiotic is re-absorbed by the intestine after first pass metabolism and returned to systemic circulation [41,42]. Cycling the toxin back through the system would redispose it in tissues, altering toxin compartmentalization dynamics.

CTX depuration in muscle tissue was reported to be rapid in recent studies of juvenile grouper, with half-lives for P-CTX-1, -2 and -3 reported as 28, 26 and 33 days, respectively [20]. Our data on C-CTX-1 depuration in pinfish muscle contrast with these results with C-CTX (as CTX3C eq.) concentrations being significantly higher on the last day than at the start of depuration (Figure 2A), highlighting possible species-level differences. Our data from day 119 likely pull the regression analyses towards a positive slope, but the low variability between replicate fish and significant differences measured between days 20 and 119 showed that an increase in CTX tissue concentration in muscle was real. Further supporting this phenomenon, it should be noted that during long-term studies such as ours, fish are growing, and if CTX levels in muscle were growth-corrected a further increase would be expected. These effects and models were comprehensively explored in this study. Decreasing trends in viscera were paralleled by the increasing CTX levels observed in muscle tissue, suggesting that CTX may be redistributed and/ or compartmentalized into muscle from other tissues through time. A biphasic change in the location of the cyanotoxin cylindrospermopsin was also reported in mussels and attributed to a re-distribution of the toxin within tissues [43]. This may explain the persistent high levels of CTX found in the upper-trophic-level fish in regions with frequent CTX exposure. Analysis of CTX levels in blood between days 90 and 119 may have determined if enterohepatic recirculation of CTX was occurring but was not performed in this study. These data remind us that toxicokinetic models of CTX should consider organ compartments as dynamic places where the toxins have the potential to be in flux and influenced by many environmental and physiological mechanisms, yet to be elucidated. Hopefully, these data will press further studies to investigate the CTX dynamics between tissues, since this may aid in fisheries management and future dockside testing once rapid field assays become available (i.e., knowing which tissues will provide a reliable assessment of CTX risk).

There were no significant trends observed in the liver over time, except for highly variable CTX concentrations throughout the depuration phase. This variability could be attributed to metabolic process regulating elimination from liver. For instance, novel glucuronide metabolites of C-CTX were recently identified in hepatic microsomes indicating that C-CTX glucuronidation may be a prevalent biotransformation pathway in fish [44]. In contrast, grouper efficiently eliminated 90% of the P-CTX from liver by the end 30 days [20]. Additionally, under controlled exposure conditions, pooled lionfish liver samples (*n* = 1 of pooled fish) analyzed by N2a-MTT gradually became less toxic over a 43 d depuration.

### 3.4. CTX Tissue Distribution

The relative CTX distribution in *L. rhomboides* tissues allowed us to compare the disposition of CTX burden across fish of different sizes through time (Figure 3). The order of highest to lowest CTX burden during the entire study was viscera > muscle > liver, but prior studies have reported a similar analysis with variable findings. The highest percentage of total CTX was in the muscle of grouper fed 1 ng P-CTX g^−1^ fish day^−1^ (approximately 50-fold higher dose than the present study) followed by other visceral tissues (intestine and liver combined) for the entire experiment (except day 2 where skin CTX burden was highest) [20]. Our visceral extract contained several tissues not analyzed in the grouper (heart, spleen, gall bladder) which have been shown containing high CTX concentrations in some fish and could explain the differences reported between studies [19]. The CTX burden reported in the muscle of mullet also contained the highest toxin load when fewer than nine CTX meals had been given [19]. However, similar to our findings, data extending beyond nine CTX feedings on dinoflagellate cells by mullet, saw most of the CTX activity detected in the intestine and gall bladder (~50%) followed by muscle (22%) and a small amount in liver (2%) [19]. Likewise, giant clams also contained most of the CTX in the viscera following exposure [36].

### 3.5. One-Compartment Model Kinetics and Growth Correction

The C-CTX-1 uptake rate calculated for the combined tissue compartments in *L. rhomboides* was 8.80 × 10^−3^ ng CTX3C eq. g^−1^ TE day^−1^. For comparison, the estimates for P-CTX-1 and -2 uptake rates in whole body of juvenile grouper were 25.2 and 3.51 × 10^−3^ ng g^−1^ day^−1^ by LC-MS analysis [20]. While our analyses do not account for CTX in some organismal compartments (e.g., kidney, carcass), the included tissues likely contained the large majority of CTX that was bioaccumulated. Toxin accumulation was lower in muscle tissue, which was exhaustively removed from fish during dissection, except for muscle tissue surrounding the head, which was difficult to reliably remove from the small fish and minor in mass. Bioaccumulation in hard parts such as bone and fin are possible but are likely minor contributors to storage and not evaluated in this study. CTX activity was below detection in the brain and gonads so these were excluded from whole body concentrations reported in this study. There is still a lot of room for future studies that will better inform organismal models of CTX bioaccumulation, and these should include longer period of bioaccumulation, different fish species, life stages, and varied CTX congeners, for a more comprehensive analysis of bioaccumulation rates.

Fish growth which contributes to lowered concentration of contaminants, can be a difficult effect to control when investigating depuration, and complicates analyses in exposures when significant [45]. Clausing et al. [33] found that naso growth masked CTX accumulation, causing an apparent steady state in muscle at 8 weeks with similar CTX concentrations occurring at 16 weeks of exposure and significant growth. In that example, growth dilution kept CTX concentrations stable between weeks 8 and 16 while the muscle CTX burden continued to increase. This effect cannot be ignored in the present study which covered a 4 month timeframe. Quantifying the growth-dilution effect has been handled in several ways when studying elimination of contaminants, including adjusting measured concentrations by a growth correction factor as performed by Li et al. [20]. However, these growth adjustment strategies have been critiqued by others and alternative strategies suggested [45,46]. In our study, we applied several growth models and corrections including the one reported by Li et al. [20] to determine the best approach for this and future investigations for C-CTX depuration and growth. We first evaluated the rate constant subtraction method outlined by Brooke and Crookes [45], which eliminates the change in concentration due to growth from the other first-order depuration process (i.e., elimination by respiration, feces, and metabolic transformation). Essential to our rate constant growth correction analyses were the predicted concentrations (*C_fish(i)_*) from body CTX burden and weight estimates at the start of depuration. In our case, knowing the feeding rate, dose, and growth rates of all fish (i.e., for *n*= 40 C-CTX treated; and *n*= 37 control) in the study allowed us to perform model analyses that have not been used in past CTX exposure studies. While first order kinetics were assumed based on regression of the transformed concentration data (Figure 4), we acknowledge that a first order relationship is approximate, and is a potential source of uncertainty in the model. However, this method did provide evidence that fish growth was the major contributor to declining CTX concentrations through time in this study.

The growth-corrected half-life for P-CTX-1, -2 and -3 (e.g., 21, 21, and 38 d, and R^2^ equal to 0.97, 0.84, and 0.37, respectively) in the whole body of the juvenile grouper *E. coioides* [20] were much shorter than reported by us and others [11,17]. This anomaly could be attributed to unequal toxicokinetics between *E. coioides* and other fish, the use of juvenile specimens, differences between CTX variants, or the statistical power or models applied in that study. Our growth correction modeling technique increased the estimated CTX depuration half-life from approximately 80 d in the raw data simulation to approximately 146 d (Figure 7A–D). Compared to other studies, our growth-corrected half-lives of total ciguatoxicity are more similar to estimates in naturally incurred ciguatoxic Pacific moray eels (*L. javanicus*) and red snapper (*L. bohar*) where half-lives of 264 and 900 d were reported, respectively [11,17]. Comparison to these data would depend on whether the fish grew significantly, but if growth was positive (as would be expected), growth correction would increase the reported half-life. From this study and others, we might expect a half-life of several months or longer which would be in line with long-term patterns of persistent ciguatoxicity in fish from regions with CTX-producing benthic dinoflagellates. Additionally, brevetoxins which are also Na_v_ activators produced by the pelagic dinoflagellate *Karenia brevis,* have been detected in fish livers more than a year after algal blooms subside [32]. The CTX binding affinity for the primary Na_v_ receptor was also shown to outcompete brevetoxin for the site [47]. Therefore, it is not unreasonable to consider CTXs have extremely long biological half-lives that are likely to vary based on the physicochemical properties of each toxin variant.

In this study of *L. rhomboides*, the calculated CTX burdens for each fish at the end of the depuration period were not measurably different than burdens measured (or estimated using the correlation between cumulative dose and total CTX burden) for day 20 (see Supplementary Figure S4). Although the level of uncertainty in the kinetic rate measurement limits the precision of our half-life estimate, the remaining high CTX burden after depuration suggests that the models presented here are valid and show that pseudo-elimination via growth was a primary factor in lowering concentration in *L. rhomboides*. The growth correction Model 1, Model 2, and Model 3 (Figure 7B–D) showed that the use of mean and individual measured growth rate constants agreed, suggesting that this method is robust enough for use in food web modeling if accurate estimates of growth rates are available for wild-fish. Age and growth studies are available for numerous reef fish species globally and a thorough example of how these data could be useful was recently provided in a critical review on conceptual models for ciguatera risk assessment [23].

### 3.6. Conclusions and Implications for Trophic Transfer

In this study we report that C-CTX-1 was bioaccumulated in *L. rhomboides* from low environmentally relevant doses (0.02 ng CTX3C eq. g^−1^ day^−1^), and present that total ciguatoxicity was retained for at least 3.3 months and depuration was mostly a function of fish growth, a pseudo-elimination factor. These data highlight that field determined concentrations of C-CTX may not reflect the mass balance of CTX that is bioavailable in food webs. Increased elimination may be expected in wild-caught fish that have varied diets and expend more energy, thus augmenting metabolic rates and lowering CTX levels. However, the retention of C-CTX-1 in *L. rhomboides* at a basal level is remarkable. This long-term CTX retention in fish helps to explain how regions with no recent sign of *Gambierdiscus* sp. blooms can produce toxic fish that cause CP outbreaks, whether by fish migration or after a period of low CTX production [12,48]. This study also suggests that field data may need to be staggered if *Gambierdiscus* abundance and toxicity are collected as a baseline in parallel with fish samples for food web toxin analysis, as is the traditional framework for field studies. Future studies could include repeated exposures with intermittent depuration periods to understand CTX kinetics once fish are re-exposed to the toxins while still depurating, which likely occurs in migratory fish and in areas with regular temporal swings in toxicity [6,9]. Quantifying the kinetics of elimination via respiration, feces, and metabolic transformations, as well as sequestration in reproductive tissues, may also be useful for future food web models of CTX exposure.

Improving our understanding of CTX kinetics has clear advantages to our interpretation of CTX dynamics in the field, and in developing improved risk models to prevent CP. Our prior efforts have shown clear pulses of C-CTX in the benthic algal community [9] in hyperendemic regions that may be dictated by lower temperature tolerance of toxigenic *Gambierdiscus* species [8,49]. If the timing of the CTX source pulses occur in higher frequency than the depuration of CTXs at higher trophic levels, we may expect increasing prevalence of toxic fishes once CTX-producing algae are established in a region. While more evidence on these dynamic cascades and factors that might influence them is needed, experimental studies such as these help to parameterize the capacity of toxin removal from the organism. From a resource management perspective, understanding periods of high and low risk, even if only possible for fish with high site fidelity, would be a great advantage to the fishery, especially when coupled to spatial resolution of CTX.

## 4. Materials and Methods

### 4.1. Reagents and Chemicals

All solvents were HPLC grade obtained from Fisher Scientific (Waltham, MA, USA) and were acetone, MeOH, *n*-hexanes, CHCl_3_, and dimethyl sulfoxide (DMSO). Bond Elut silica solid-phase extraction (SPE) cartridges (100 mg and 500 mg) were from Agilent Technologies (Santa Clara, CA, USA). Ouabain octahydrate (O) and veratrine hydrochloride (V) used for in vitro assays were from Sigma (St. Louis, MO, USA). The 3-[4,5-dimethylthiazol-2-yl]-2,5-diphenyl- tetrazolium (MTT) was sourced from Alfa Aesar (Haverhill, MA, USA) and was prepared in sterile phosphate buffered saline (PBS) from Medicago (Quebec City, QC, Canada). Ciguatoxin 3C (CTX3C) was purchased from Wako Chemicals (Osaka, Japan) and a 50 ng mL^−1^ stock was prepared in LCMS grade MeOH and aliquoted in sealed amber vials maintained at −20 °C until use.

Adherent murine neuroblastoma cells (Neuro-2a; ATCC CCL-131) were originally purchased from the American Tissue Culture Collection (Manassas, VA, USA) and modified (i.e., OV desensitized) lines generated and maintained to ensure maximal and stable cell response to CTX in MTT-based assays prior to use (available on request). Powdered Roswell Park Memorial Institute (RPMI) 1640 medium (Millipore Sigma, Burlington, MA, USA) was prepared in 10 L batches with sterile ultrapure water (18 mΩ) and 1L aliquots filtered (polyethersulfone 0.2 µM Supor membrane; Pall Corp; Port Washington, NY, USA) into sterile bottles. Supplements included sterile L-glutamine (200 mM stock), sodium pyruvate (100 mM stock), and fetal bovine serum (all from Gibco; Grand Island, NY, USA). Trypsin-EDTA (0.025% stock) used in cell detachment and harvest was from Corning (Corning, NY, USA). Cell culture consumables including serological pipettes, tubes, flasks, and micro-well plates were from CellTreat (Shirley, MA, USA). Trypan blue (Fisher Scientific, Waltham, MA, USA) was prepared to 0.2% in sterile PBS (pH 7.4) and used in cell enumeration and viability assessment.

### 4.2. Controlled Exposure

#### 4.2.1. Fish Collection and Acclimation

Wild pinfish, *Lagodon rhomboides* (Sparidae), were collected from Mississippi Sound (Dauphin Island, AL, USA) and Perdido Bay (Orange Beach, AL, USA) using hook-and-line with sabiki rigs. To reduce stress from handling, fish were directly transferred to an aerated, seawater-filled cooler using a specialized dehooking tool which does not require fish handling. Fish were transported in aerated, temperature and salinity-controlled tanks to the experimental wet-lab facility at the Dauphin Island Sea Lab (DISL) within one hour. Prior to transfer to a primary acclimation tank, a five-minute freshwater dip was performed to remove marine ectoparasites. This step was important to reduce the chance of disease in the closed system. The recirculating aquarium system was composed of a 450 L sump, 375 L head tank, and 120 L enclosure tank and filled with artificial seawater (Crystal Sea Marine mix; Mount Dora, FL, USA). To limit stress, environmental enrichment provided to each tank habitat included a two-inch bed of pool filter grade silica sand, 12 cm diameter × 12 cm length PVC tubes for structure, and artificial submerged aquatic vegetation. Fish were collectively monitored in the acclimation tanks in groups of 25 for three weeks for signs of parasitic diseases and behavioral abnormalities prior to transfer into individual tanks for experimental treatment. Two fish collections were performed to supply fish for the exposure study, once in January 2019 and again in May 2020. The water conditions across all systems were maintained on a 12 h light: 12 h dark cycle with weekly water changes (artificial seawater: 15–17 psu; 24 ± 2 °C; ammonia ≤ 0.1 ppm, nitrite ≤ 0.1 ppm, nitrate ≤ 40 ppm, pH = 8.1 ± 0.1).

#### 4.2.2. Feed Formulation

Experimental (CTX feed) and control diets (no CTX) were created from a blend of fish meal, Mazuri Aquatic Gel Diet for Omnivorous Fish (sku: 1815252-409; Mazuri Exotic Animal Nutrition, St. Louis, MO, USA), and water. The fish meal consisted of white muscle tissue from either non-toxic great barracuda, *Sphyraena barracuda,* collected from the northern Gulf of Mexico, Alabama (control) or *S. barracuda* with naturally incurred C-CTX that were collected from the U.S. Virgin Islands during concurrent efforts. Fillets with skin, scales, and bones removed, were cut into small chunks and subsequently homogenized in an industrial food-grade stainless steel grinder (STX Turboforce 3000; Lincoln, NE, USA). From each fish, at least five replicate subsamples of minced *S. barracuda* tissue were extracted and analyzed for the presence of CTX-like activity using N2a-MTT, and C-CTX-1 confirmed by liquid chromatography-mass spectrometry as previously reported [50]. After verifying control and CTX fish, batches were pooled and mixed three times, then frozen in stainless steel trays and freeze dried. Dehydrated tissue (300 g) was powdered using a grain mill (HC-300; C-Goldenwall, Amazon, Seattle, WA, USA) at 28,000 rpm for 1 min and sieved to ≤500 µm to create a fine and uniform product. All fish powder was thoroughly mixed and kept in 500 g aliquots at −20 °C in airtight containers until use. Subsamples of the homogenized freeze-dried fish powder were taken for analysis by the N2a-MTT for final quantification of toxicity of the pelleted diet prior to further preparation. The control and CTX diets for *L. rhomboides* were created by combining equal parts Mazuri gel powder and fish powder, then homogenized and combined with 60% water (by weight) that was warmed to 65 °C. The homogenized feed was transferred to a pastry piping bag with a round tip (Wilton size 10), then dispensed in even rows onto pre-weighed parchment paper. After fully dispensing the gel, wet weight was recorded, and the gel was cut into 5 mm pellets. The tray was then placed in a drying oven at 65 °C for approximately 1 h to remove approximately 2/3 water so that pellets retained a uniform shape. To maintain a consistent product across batches, the water content (as a percent of the final product) was calculated by subtracting the weight of solid ingredients in the mixture from the weight of the formulated food after drying, then dividing by the whole-food dry weight. Subsamples of pellets were weighed on an analytical balance to collect an average pellet weight.

#### 4.2.3. Experimental Design

To accomplish optimal control and observation of fish, *L. rhomboides* were individually transferred to 12 L tanks attached to a closed recirculating aquarium system. Four recirculating aquarium systems with 10 to 15 tanks per system were used in this study. Fish were acclimated to the individual tanks (one fish per tank) for at least 2 weeks prior to the beginning of experimental treatment to ensure water quality parameters and fish health were stable. This acclimation period was in addition to the post-collection acclimation.

The experiments were designed to provide control over dietary intake of C-CTX-1 in individual fish so that the toxicokinetics of bioaccumulation and depuration could be analyzed across multiple tissues with sufficient replication for the subsequent statistical analyses. Tanks designated to receive control or CTX pellets were assigned a sampling day on the day prior to the start of experiments using a random number generator. Control fish received control pellets throughout trials while CTX fish received CTX pellets for up to 20 days (bioaccumulation phase) and then transitioned to the control feed for the depuration phase (see Table 1 for sampling scheme). Both control and CTX pellets contained powderized *S. barracuda* flesh (non-toxic or toxic, respectively) to match the nutritional intake across groups and maintain consistency (minus CTX) when fish were transitioned from the bioaccumulation to the depuration phase. Fish fed only the control pellets were designated for each sampling point to be used as matrix controls in assays, and to compare differences in behavior, and growth rate compared to the CTX treatment groups.

Data were collected from two feeding experiments (trial 1 and 2, hereafter) to allow adequate replication. The first experiment had maximum bioaccumulation and depuration phases of 20 days each with a total of 52 fish and was performed 5 March–13 April 2019. Fish were sampled as baseline controls prior to feeding on the first experimental day and on days 6, 10, and 20 of bioaccumulation and days 5, 10, and 20 of depuration. In the second experiment performed 30 July–25 November 2020, the bioaccumulation phase was replicated identical to trial 1, and depuration was extended out to a maximum of 99 days to increase the depuration course. Fish were collected on day 20 of the bioaccumulation phase to compare to trial 1 for reproducibility, and then on days 40, 70, and 99 of the depuration phase. Based on the lack of overt signs of intoxication during the first experiment and ample analytical controls, during the second experiment, remaining *L. rhomboides* were allocated towards maintaining experimental replication in the CTX treatment group (CTX fish, *n* = 4) and control replicates were reduced to *n* = 2 per time point (except day 0, *n* = 3) (Table 1).

Daily feed requirements were calculated based on initial weights (g) of fish which were recorded using a water displacement method one or two days prior to the beginning of exposure trials. All fish were fed either control or CTX pellet food at the same time daily, normalized to body mass (approximately 1.8% of initial body weight day^−1^). Fish were fed the same amount of food daily relative to the initial whole wet weight for the entire experiment. Individuals were closely observed to ensure all food was eaten during feeding times, and after acclimation *L. rhomboides* were consistently consuming the pellets within seconds of entering the tank. Fish were also re-weighed at multiple points (at minimum initial and at time of sampling) throughout the study to track growth rate and to determine if somatic growth influenced C-CTX concentration as previously expected by others in P-CTX fish studies [20,33,51].

#### 4.2.4. Fish Sampling, Dissection, and Extraction

On a designated day and time of sampling, fish were fed and left undisturbed for 7 h. Fish were euthanized according to approved IACUC protocols by iced seawater immersion and mortality was confirmed by cessation for at least 10 min. A secondary spinal transection was performed to ensure mortality, and fish were then dissected. Dissected tissues were weighed whole including muscle, brain, liver, gonads (when present), and additional visceral organs combined (heart, spleen, pancreas, gall bladder, intestine). Swim bladder was discarded and not analyzed in this study. Stomach was removed from the viscera samples to limit residual CTX signal from undigested food in downstream toxicity assays; however, given reported gut clearance rates <24 h, the amount of residual CTX in the intestinal tract was expected to be below the limit of quantification (LOQ; described in Section 4.3 Data Analysis) in the viscera on our assays.

Muscle subsamples (5–7 g) were extracted twice in acetone (2 mL g^−1^ tissue weight) by bead disruption (2.6 mm diameter; ceramic) using a Bead Ruptor 24 (Omni International; Kennesaw, GA) at a speed of 5 m/s for two cycles of 30 s duration each. Resultant homogenates were centrifuged at 2465× *g* for 5 min at room temperature (approximately 21 °C) between extractions to obtain the supernatant. Combined supernatants were placed at −20 °C for 18 h, then centrifuged (4 °C, 2465× *g*, 10 min), and supernatants dried under a gentle nitrogen stream (45 °C). Dried residues were reconstituted in 90% aq. MeOH (1 mL g^−1^ original weight) and partitioned twice using *n*-hexane (2 mL g^−1^ original weight) to remove non-polar lipids. The aq. MeOH phase was dried under nitrogen, and the resultant residue was partitioned with CHCl_3_: H_2_O (50:50, v:v). The CHCl_3_ layer containing CTX was collected, then water phase partitioned again with the same volume of CHCl_3_. Pooled CHCl_3_ fractions were dried, reconstituted in 500 µL CHCl_3_, and further cleaned by silica SPE. Powderized fish tissue (CTX and control) was extracted in a similar manner using a 5:1 solvent (mL) to tissue (g) weight ratio. Non-muscular tissues were extracted whole due to their small size and solvent ratios adjusted accordingly. Final tissue extracts were dissolved in 1 mL 100% MeOH and stored at −20 °C until analysis.

### 4.3. Toxin Analysis

#### 4.3.1. Maintenance of Neuroblastoma Cells

Cells were maintained in vented 175 cm^2^ sterile culture flasks with RPMI medium supplemented with 5% heat-inactivated FBS, 1 mM sodium pyruvate, and 2 mM L-glutamine (complete media) and maintained in a humidified water jacketed incubator at 37 °C with 5% CO_2_: 95% atmospheric air. Cells were passaged every 48 h and maintained in exponential growth. During passaging and in preparation for seeding 96-well plates, cells were harvested with 0.025% trypsin-EDTA for <2 min, trypsin deactivated with 10% FBS-RPMI complete medium, centrifuged, supernatant discarded and cells washed two times in PBS. Duplicate aliquots of cells resuspended in 5% FBS-RPMI medium were enumerated on a hemocytometer to calculate growth rates, and passage % viability via trypan blue staining.

#### 4.3.2. Neuroblastoma MTT Assay (N2a-MTT)

Sample extracts were tested for composite voltage-gated sodium channel (Na_V_) response using a standardized in vitro mouse neuroblastoma assay (N2a-MTT) as previously described [50] with toxin quantification of serially diluted samples evaluated and compared to a 9-point 2-fold serial dilution of CTX3C (initial dose equal to 20 pg well^−1^ or 86.96 pg mL^−1^). The European Food Safety Authority (EFSA) has developed guidelines outlining toxic equivalency factors for the various CTX-group toxins [52]. CTX3C has been reported to be two-fold more toxic than C-CTX-1 based on intraperitoneal toxicity and was accepted here as the better certified reference standard compared to P-CTX1 which was reportedly ten-fold more toxic than C-CTX-1. The difference in CTX3C standard toxicity could cause lower estimates of C-CTX-1 content. For better understanding of toxin content, conversion from CTX3C eq. to C-CTX-1 can be done by multiplying CTX3C eq. concentrations by a factor of 2.

Due to the sample size of subsampled fish tissues, we were unable to perform additional LC-MS/MS analysis which requires much higher CTX concentrations compared to N2a-MTT. These analyses could have been accomplished by pooling tissues and/or extracts from replicate fish for each sampling point as described by others [20] but this would have lost the experimental replication that we deemed critical to the validity of this study, so was delayed to a future study with larger fish specimens.

N2a cells from established OV adapted lines, were seeded into 96-well plates at a density of 3 × 10^5^ cells per well, in complete RPMI media (200 µL). After 20 h, cells were dosed in triplicate with standards, controls, and sample extracts; all with and without OV. To prepare fish extracts for dosing, an extract aliquot dissolved in MeOH was transferred to a 1.5 mL microcentrifuge tube, dried under ultrapure N_2(g)_, and redissolved in 5% FBS-RPMI complete media by vortex (30 s, room temperature). Cells in assay wells were carefully inspected by light microscopy prior to dosing and development. Positive controls (CTX-positive reference material), negative controls (containing PBS and medium), and assay controls with (sensitized) and without (non-sensitized) O/V (final well concentration: 0.22 mM ouabain/0.022 mM veratrine) were used to ensure quality assurance and control throughout the several hundred assays performed during this study. Sample wells were dosed with 10 µL of the fish extract solubilized in culture media or PBS (final well volume 230 µL). After a 20 h incubation, well contents were removed and MTT (1 mg/mL) diluted in 5% FBS-RPMI-1640 complete medium was added for 30 min. The resultant insoluble formazan product produced by mitochondrial activity of remaining live cells was solubilized in 100% DMSO (100 µL) with the colorimetric change measured within 5 min on a spectrophotometric microplate reader (µQuant; Biotek Instruments; Winooski, VT, USA) at 570 nm. Cells sensitized with O/V were used to assess CTX-like activity, while non-O/V-sensitized cells were used to monitor non-specific activity induced by sample extracts. When O/V-dependent toxicity was detected by at least a 20% difference between controls and wells dosed with fish extracts, a two-fold serial dilution of extract was prepared and assayed parallel to a CTX3C standard dilution series on the same day.

#### 4.3.3. N2a-MTT Data Analysis

Raw data were analyzed using Microsoft Excel version 2013 (Microsoft Corporation, Redmond, WA, USA). Normalized data were analyzed with GraphPad Prism version 9.0.0 (GraphPad Software, San Diego, CA, USA). 

Raw absorbance values for wells dosed with standards and fish extracts were normalized to the OV control wells (20 µL O/V + 10 µL PBS, or 30 µL PBS), to account for minor OV N2a mortality established during cell line adaptation as described by others [53]. Triplicate absorbance responses within a single assay plate were deemed acceptable when the relative standard deviation was below 20%. To produce standard curves for quantification on the day of each assay, CTX3C standard doses (x-values) were logarithm transformed and fit by non-linear regression against the normalized response (y-values) in wells at each dose using a four-parameter logistic equation with variable slope (Y = Bottom + (Top/Bottom)/(1 + 10^((LogIC_50_ − X) ∗ HillSlope). Toxicity of tissue extracts were estimated by interpolating the normalized responses (y-value) in wells onto the standard curve to estimate the dose (unknown x-value) in pg CTX3C equivalence. Values that fell between the effect concentration 20–80 (EC_20_ to EC_80_) which is the linear portion of the standard curve are acceptable, but we chose to use more strict parameters for quantification by accepting only values between EC_30_ and EC_75_ because full curves with a top and bottom plateau were not always possible based on the CTX concentrations in tissues. Interpolated results (pg CTX3C eq.) were divided by the tissue equivalence (mg TE) of the dose to calculate the concentration in ng CTX3C eq. g^−1^ TE. The LOQ for CTX in each tissue type was determined by dividing the mean value of the EC_75_ from the CTX3C dose–response curve and the maximum TE dosed in wells without a matrix induced effect defined as either growth enhancement >20% of the control wells or cell death associated with non-Na_v_ mechanisms (evaluated in sample wells without OV). All samples were analyzed in triplicate across 2–4 independent assays performed on separate days with a CTX3C standard curve prepared on the day of each assay.

### 4.4. Ciguatoxin Kinetics

#### 4.4.1. Muscle, Liver, and Viscera Ciguatoxin Kinetics

Kinetics of CTX uptake and depuration in separate tissues were investigated through non-linear regression analysis of the experimentally determined CTX concentration in sample extracts against time for both phases using GraphPad Prism version 9.0.0 (model comparison function). When CTX was below detection levels due to a non-specific matrix effect, those replicates were excluded from analyses (see Results Section 2.5). Normality of toxin data was evaluated using a Shapiro–Wilk test and models were fit using a least squares regression with no weighting and compared by an iterative process. Best fit models were compared based on the Akaike’s Information Criterion (AICc) which balances the goodness of fit using sum-of-squares and the simplicity (number of degrees of freedom) of the two models.

#### 4.4.2. One-Compartment Model Kinetics

The combined CTX concentrations (*C_fish_*) for the measured tissues were calculated for each fish by multiplying the quantified concentration for each tissue (muscle, liver, and viscera) by the whole tissue mass (conc. x mass = tissue burden), summing the tissue CTX burdens, and dividing by the total sum of the whole muscle, liver, and viscera mass. The *C_fish_* was used to investigate a one-compartment model of CTX kinetics which assumes a homogenous concentration in the fish. While our analyses do not account for CTX in some compartments (kidney, stomach, and carcass), the included tissues (muscle, liver, viscera) contained the majority portion of CTX in the major tissue compartments in the fish. No CTX activity was detectable in brain or gonads, so these were excluded.

The uptake rate was calculated by linear regression of the measured *C_fish_* (ng g^−1^) during the bioaccumulation phase against time (d = 0 to 20)

(1) $$C_{fish}=k_{uptake}\times t+a$$

where the slope of regression is equal to the rate of increasing CTX concentration (*k_uptake_*; ng g^−1^ day^−1^), *t* is time (d), and *a* is a constant which in this case is the y-intercept (ng g^−1^).

The first-order depuration rate constant (*k*_2_) was calculated using the methods outlined by Brooke and Crookes [45]. A one-compartment model of exponential decay was fitted to the measured *C_fish_* during the depuration phase

(2) $$C_{fish\left(t\right)}=C_{fish\left(i\right)}\;e^{-k_{2}t}$$

where *C_fish(t)_* is the concentration (ng g^−1^) measured at the time of sampling, *C_fish(i)_* is the initial concentration (ng g^−1^) at the start of the depuration phase (day 20), *t* is time (d), and *k*_2_ is the overall depuration rate constant (day^−1^). For curve fitting, the measured *C_fish_* in the depuration phase were natural Log transformed, i.e., Ln [*C_fish_*], to allow linear regression of Log-concentrations versus time in which the slope of regression is the *k*_2_, and the coefficient of determination is used to confirm first-order kinetics. The *k*_2_ calculated by these methods is the overall elimination rate constant including the sum of four first-order kinetic processes

(3) $$k_{2}=k_{r}+k_{m}+k_{e}+k_{g}$$

where *k_r_*, *k_m_*, and *k_e_* (all in units day^−1^) are the rate constant for elimination via respiration, metabolic transformation, and feces, respectively, and *k_g_* is the rate constant for the change in concentration due to fish growth, a pseudo-elimination process. For an in-depth summary on each of the rate constants and application to overall *k*_2_, see Gobas [54] (pp. 4–9).

To correct the overall *k*_2_ for growth of the fish, *k_g_* was calculated using the growth rate data collected for each fish. Fish masses were applied to an exponential growth model

(4) $$W_{fish\left(t\right)}=W_{fish\left(i\right)}e^{k_{g}t}$$

where *W_fish(t)_* is the wet weight (g) of the fish at any point, *W_fish(i)_* is the initial wet weight at the start of the experiment, and *k_g_* is the growth rate constant (day^−1^). To allow a linear fit, the inverse of measured weights was natural Log transformed, i.e., Ln (1/*W_fish_*), and plotted against time where the slope is equal to *k_g_* (day^−1^). The effect of growth, which results in diluted concentrations over time, was factored out of the depuration rate constant by subtracting the *k_g_* from the overall *k*_2_ to give the growth-corrected depuration rate constant (*k*_2 *growth-corrected*_; day^−1^)

(5) $$k_{2growth-corrected}=k_{2}-k_{g}$$

and describes the rate constant for elimination processes that result in removal of CTX from the fish (*k_r_* + *k_m_* + *k_e_*) [45].

#### 4.4.3. Kinetic Modeling and Correction of Growth Dilution

The exponential growth Equation (4) was used to estimate the mass of each fish on day 20 (end of bioaccumulation phase) that entered the depuration phase using measured growth rate constants (*k_g_*) and solving for *W_f(t)_*. For trials 1 and 2, respectively, values of *t* were set to 20 and 22 days, because initial weights were collected on day 0 (trial 1) and two days prior to start (trial 2), while depuration was initiated on day 20 for both trials. The sum of muscle, liver, and viscera mass was estimated at day 20 using a linear correlation between whole fish mass (g) and the sum of tissue masses, i.e., Σ (whole muscle + liver + viscera; g), that were dissected from sampled fish for CTX analysis. The combined CTX burden in the whole muscle, liver, and viscera at day 20 was estimated from the linear relationship between the cumulative dose administered (total ng CTX3C eq. consumed = number of pellets fed daily × number of days in bioaccumulation phase × pellet CTX3C eq. conc.) and the combined CTX burden measured in fish that were sampled between 0 and 20 d. Estimates of total CTX burden and combined tissue mass at the end of the bioaccumulation phase (day 20) were divided for an estimate of an overall CTX concentration in the fish at the start of depuration (*C_fish(i)_*).

A simulation model of our data was created using the estimated *C_fish(i)_* to study the effect of fish growth on elimination of CTX. Final concentrations were simulated for each fish in the depuration phase at all time points using the one-phase exponential decay model (Equation (2)) where *C_fish(i)_* is an estimate on day 20, time is delta time in depuration depending on when the fish was sampled, and overall *k*_2_ was measured from the linear plot depuration data. The resulting simulated concentrations were natural Log transformed (as previously described for the measured *C_fish_*) and plotted against time (d) to compare the regression slope (*k*_2_) with the slope from the measured data. A correlation was then performed to check how well the simulated data fit the measured concentrations.

Simulated concentration data served as the baseline for growth-corrected models to compare the change in half-life (Ln [2]/ *k*) under different conditions of *k_2 growth-corrected_*. The growth-corrected models were produced by adjusting the depuration rate constant (*k*_2_) in the simulation exponential decay model. Three growth-corrected models were produced using different values of *k*_2 *growth-corrected*_ that were calculated based on fish growth rates in the depuration phase, i.e., average growth rates at each sampled time point (Model 1), average growth rate based on ANOVA grouping (Model 2), and the individually measured growth rate (Model 3). Results of each model compared to the simulation are presented using the linear version of the exponential decay model where concentrations are natural Log transformed and plotted against time. The growth-corrected half-lives of the models were finally compared to the half-life calculated from growth correction by a simpler approach reported by others [20,55] that uses a growth correction factor, where measured concentrations in fish during the depuration phase are multiplied by (1 + *k_g_* × time).

### 4.5. Statistical Analyses

Fish size (mass in g) at the initial time point across studies was analyzed using a non-parametric two-tailed Mann–Whitney U test since trial 1 weights were not normally distributed. Growth rate constants (*k_g_*) of control and treatment groups collected at the same time point were tested for normality using a Shapiro–Wilk test and compared for differences using a two-tailed *t*-test, respectively. Growth rate and toxicity data between control and exposure treatments were tested for normality and homoscedasticity using a Shapiro–Wilk test and Brown-Forsythe test, respectively, and residual plots were visually inspected as a parallel method prior to one-way ANOVA with Tukey’s multiple comparisons test. Growth trends were investigated by a linear regression plot of *k_g_* against time. A simple correlation was used to analyze the fit of the measured and model simulated *C_fish(t)_* in the depuration phase prior to investigating growth correction of the model.

## Figures and Tables

**Table 1:** Number of replicates (*n*) Control and ciguatoxin (CTX)-exposed *Lagodon rhomboides* sampled during two trials where CTX-exposed individuals were fed pelleted Caribbean CTX-1 at 0.02 ng CTX3C eq. g^−1^ fish (initial weight) day^−1^. Days in parentheses are number of days in the depuration phase while non-parentheses numbers are total days in the experiment.

		Trial 1 (N)		Trial 2 (N)	
Phase of Experiment	Experiment Day	Control	CTX	Control	CTX
Bioaccumulation	0	4	-	3	-
	6	4	4	-	-
	10	4	4	-	-
	20	4	4	2	4
Depuration	25 (5)	4	4	-	-
	30 (10)	4	4	-	-
	40 (20)	4	4	-	-
	60 (40)	-	-	2	4
	90 (70)	-	-	-	4
	119 (99)	-	-	2	4
Total		28	24	9	16

**Table 2:** Quality control of experimental treatments using the formulated pellet diet. Values are provided as the mean ± standard deviation (s.d.) of subsamples from each batch (per trial, control = 4, CTX = 3).

Quality Control Parameter	Trial 1		Trial 2	
	Treatment			
	Control	CTX	Control	CTX
Pellet Water Content (%) ^a^	35.8 ± 0.5	27.0 ± 4.0	37.3 ± 1.5	33.7 ± 2.9
Pellet Weight (g) ^b^	0.10 ± 0.00	0.09 ± 0.01	0.09 ± 0.01	0.09 ± 0.01
Food CTX Concentration (ng g^−1^) ^c^	0	1.06 ± 0.06	0	0.96 ± 0.04
Pellet CTX Concentration (ng pellet^−1^) ^c^	0	0.096 ± 0.020	0	0.091 ± 0.019
% of Initial Weight Fed Daily (whole) ^d^	1.9 ± 0.2	1.8 ± 0.2	1.7 ± 0.1	1.8 ± 0.1
% of Initial Weight Fed Daily (dry) ^e^	1.2 ± 0.1	1.3 ± 0.1	1.1 ± 0.1	1.2 ± 0.0
Consumption Rate (ng g^−1^ fish day^−1^) ^f^	0	0.019 ± 0.002	0	0.017 ± 0.001

^a^ Calculated from the weight of dry ingredients in the whole food after drying. ^b^ Calculated from the mean weight of 40 pellet subsamples per batch. ^c^ Calculated using the CTX3C eq. concentration of dried toxic barracuda powder and amount of powder in the mixture. ^d^ Calculated from the average weight of pellets provided daily and weight of fish on day 0. ^e^ Calculated using pellet water content and percentage of initial weight fed daily (whole). ^f^ Calculated from total weight of pellets provided daily, pellet CTX3C eq. concentration, and initial weight of fish.

**Table 3:** Between-trial reproducibility of CTX-1 concentrations measured as CTX3C eq. by mouse neuroblastoma assay (N2a-MTT) in fish tissues collected at day 20. Data for each trial are the mean ± s.d. (*n* = 4). All controls were negative and are not shown. A two-tailed *t*-test was performed to test for significant differences (α 0.05; df = 6).

Tissue	CTX3C Eq.		Statistics	
	Trial 1	Trial 2	*t*	*p*-Value
Muscle	0.04 ± 0.02	0.06 ± 0.01	1.39	0.214
Liver	1.01 ± 0.99	0.91 ± 0.48	0.165	0.875
Viscera	4.40 ± 1.17	3.80 ± 0.37	0.971	0.369

**Table 4:** Results of linear regression analysis for CTX3C eq. concentrations in fish samples during the experimental bioaccumulation and depuration phases.

Tissue	Statistical Parameters	Bioaccumulation	Depuration
Muscle	Linear equation	y = 0.0034x − 0.0200	y = 0.0005x + 0.0201
	R^2^	0.70	0.43
	*p*-value	0.0001	0.0005
Liver	Linear equation	y = 0.0630x − 0.3024	y = 0.0063x + 0.5277
	R^2^	0.24	0.09
	*p*-value	0.1291	0.1518
Viscera	Linear equation	y = 0.1239x + 1.704	y = −0.0331x + 6.093
	R^2^	0.47	0.43
	*p*-value	0.0034	0.0005

**Table 5:** Growth-corrected depuration rate constants (*k*_2 *growth-corrected*_) that were used in growth-dilution correction Model 1 of this study. The mean *k_g_* (*n* = 4) of replicate fish at each time point were used for the correction in this model.

Experiment Day	Trial	Average *k_g_* × 10^−3^ (day^−1^)	*k*_2 *growth-corrected*_ × 10^−3^ (day^−1^) ^a^
20	1	7.615 ± 1.492	−0.454
20	2	8.402 ± 0.345	−1.241
25	1	7.747 ± 0.676	−0.586
30	1	7.037 ± 1.132	0.124
40	1	6.489 ± 1.270	0.672
60	2	5.187 ± 0.943	1.975
90	2	4.447 ± 0.878	2.714
119	2	4.619 ± 0.567	2.542

^a^ Calculated by subtracting average *k_g_* from the overall *k*_2_ value of 7.16 × 10^−3^.

**Table 6:** The *k*_2 *growth-corrected*_ that were used in the growth-dilution correction Model 2 of this study. Time intervals where significant differences were detected by ANOVA and a Tukey’s test (See Supplementary Figure S2B) were used for correction in this model.

Experiment Day	Trial	Average *k_g_* × 10^−3^ (day^−1^)	*k*_2 *growth-corrected*_ × 10^−3^ (day^−1^) ^a^
20–25	1, 2	7.921	−0.760
30–40	1	6.763	0.398
60–119	2	4.785	2.410

^a^ Calculated by subtracting average *k_g_* from the overall *k*_2_ value of 7.161 × 10^−3^.

## Data Availability

The data in this study are available in this article and Supplementary Materials.

## Supplementary Materials

The following are available online at https://www.mdpi.com/article/10.3390/toxins13110774/s1; Supplementary Figure S1: Representative in vitro dose–response curves from the neuroblastoma-MTT assay in this study. Supplementary Table S1: Results of a *t*-test on the first order growth rate constants (*k_g_*) between Control and CTX-treated *L. rhomboides* at each sampled time point. Supplementary Figure S2: Results of ANOVA with Tukey’s test on *k_g_* of Control and CTX feeding treatments across time. Supplementary Figure S3: Results of ANOVA with Tukey’s test for C-CTX-1 measured in CTX3C eq. concentrations (mean ± s.d.) in muscle, liver, and pooled viscera of *L. rhomboides* in this study. Supplementary Figure S4: Sum of total C-CTX burdens (measured as CTX3C eq.) in the tissues of *L. rhomboides* sampled in this study.
