# Supplementary material for: Depuration Kinetics and Growth Dilution of Caribbean Ciguatoxin in the Omnivore Lagodon rhomboides: Implications for Trophic Transfer and Ciguatera Risk
Source: Toxins (Basel). 2021 Nov 1;13(11):774. doi: 10.3390/toxins13110774 (PMC8623479; doi:10.3390/toxins13110774)
Supplement: Supplementary file 1 [file toxins-13-00774-s001.zip › toxins-13-00774-s001/Toxins_Supplementary Materials_CTX Depuration in Pinfish_Resubmission final.pdf]

Supplementary Materials

Title: Depuration kinetics and growth dilution of Caribbean CTX in the omnivore Lagodon rhomboides: Implications for trophic transfer and ciguatera risk

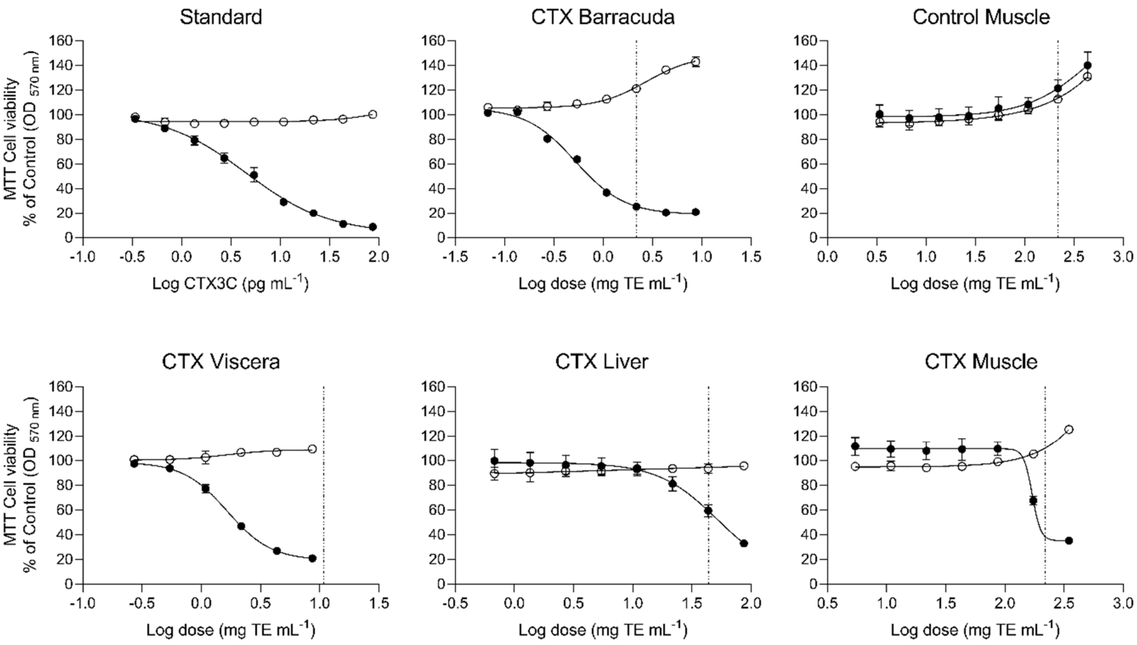

Figure S1. Representative in vitro dose-response curves from the neuroblastoma-MTT assay (N2A-MTT) when exposed to increasing CTX concentrations under conditions including ouabain and veratrine (OV) (closed circles) at 0.22/ 0.022 mM final concentration and when not including OV (open circles). The CTX3C standard, freeze-dried *Sphyraena barracuda* flesh used in the formulated Caribbean ciguatoxin (C-CTX-1) diet, and *Lagodon rhomboides* tissue extracts after Control and CTX feeding are shown. Data represent the mean ± standard deviation (s.d.) of each condition in triplicate. Absorbance (e.g., optical density (OD)) was measured using the MTT assay at 570 nm and values were normalized to control wells on the same plate under the same OV conditions minus sample extract. The mean ± s.d. of the standard EC_{50} across all assays was 4.54 ± 1.51 pg mL^{-1} (or 1.04 ± 0.35 pg $well^{-1}$). The limit of quantification (LOQ) for each tissue was set by dividing the mean EC_{75} of the standard dose-response curve (1.97 ± 0.66 pg mL^{-1}) and the maximum tissue equivalent (TE) extract dosed in wells without a matrix-induced effect (2.17, 217.4, 43.5, 10.9 mg TE mL^{-1} for *S. barracuda* flesh and pinfish muscle, liver, and viscera, respectively). The LOQ (dashed line on graph) for the barracuda flesh and pinfish muscle, liver, and viscera were 0.90 ± 0.30, 0.01 ± 0.00, 0.05 ± 0.02, and 0.18 ± 0.06 ng CTX3C eq. g^{-1} TE tissue, respectively.

Table S1. Results of a t-test on the first order growth rate constants (k_g) between Control and CTX-treated *L. rhomboides* at each sampled timepoint.

Phase of Experiment	Experiment Day	Trial	$k_g \times 10^{-3}$ (d^{-1})		Statistics		
			Control	CTX	t	df	p-value
Bioaccumulation	6	1	7.47 ± 3.26	4.51 ± 4.91	0.97	5	0.377
	10	1	6.87 ± 2.78	6.22 ± 2.94	0.32	6	0.760
	20	1	11.5 ± 2.27	7.62 ± 1.49	2.82	6	0.030
	20	2	3.64 ± 0.34	8.40 ± 0.35	16.1	4	<0.0001
Depuration	25	1	7.31 ± 2.10	7.75 ± 0.68	0.397	6	0.705
	30	1	5.59 ± 0.85	7.04 ± 1.13	2.05	6	0.086
	40	1	5.72 ± 0.16	6.49 ± 1.27	0.750	6	0.482
	60	2	5.07 ± 1.04	5.19 ± 0.94	0.135	4	0.899
	90	2	NA [a]	4.45 ±0.88	NA [a]	NA [a]	NA [a]
	119	2	4.07 ± 0.90	4.62 ± 0.57	0.961	4	0.391

[a] No controls were sampled on day 90 (NA = not applicable)

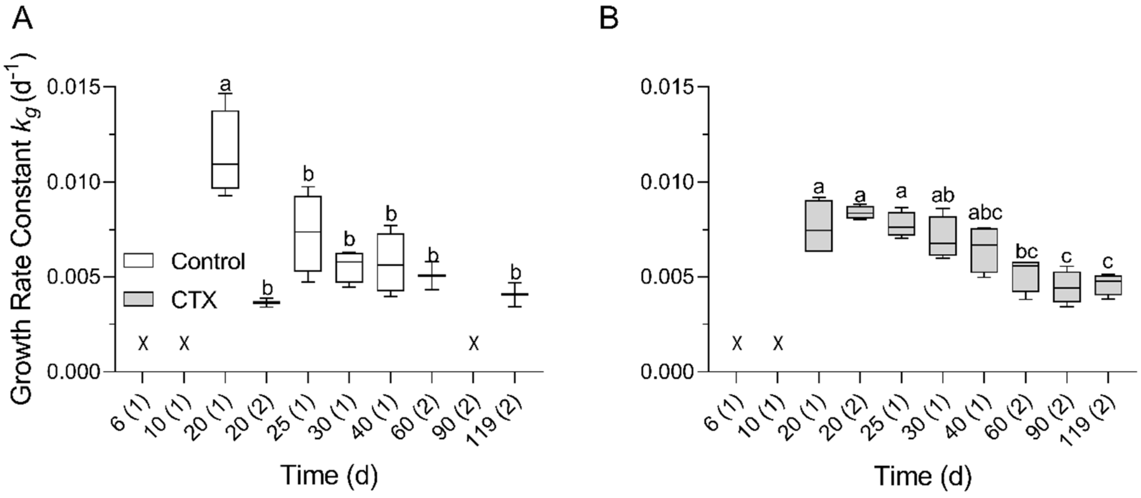

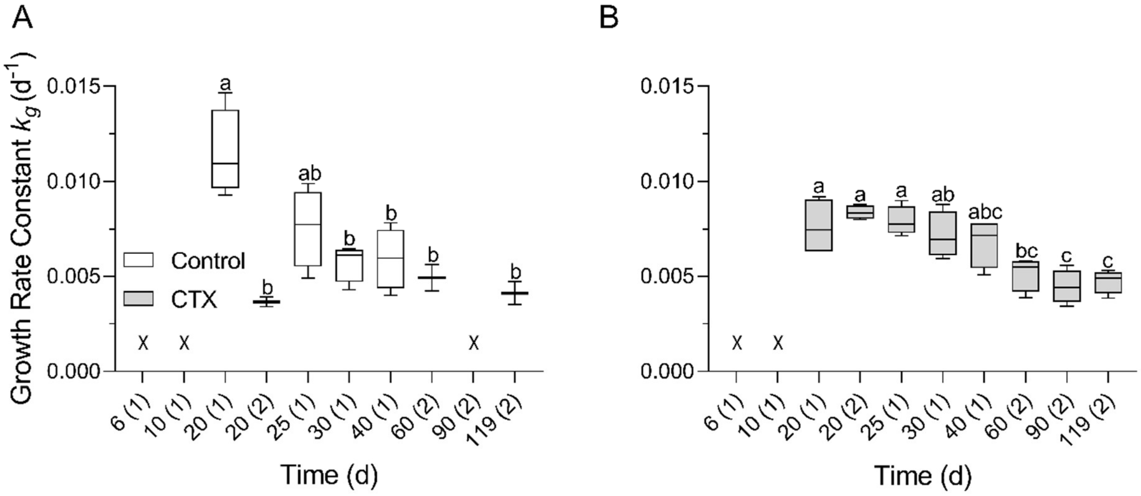

Figure S2. Results of ANOVA with Tukey's test on k_g of (A) Control and (B) CTX feeding treatments across time. Letters denote significant differences within treatment groups ($p < 0.05$). Numbers along the x-axis indicate the sampling day and trial (in parentheses; trial 1 or 2). The X symbol is where data were excluded on days 6 and 10 (see Methods) and no controls were sampled on day 90. Boxes and whiskers represent the minimum, maximum, and mean (n = 4).

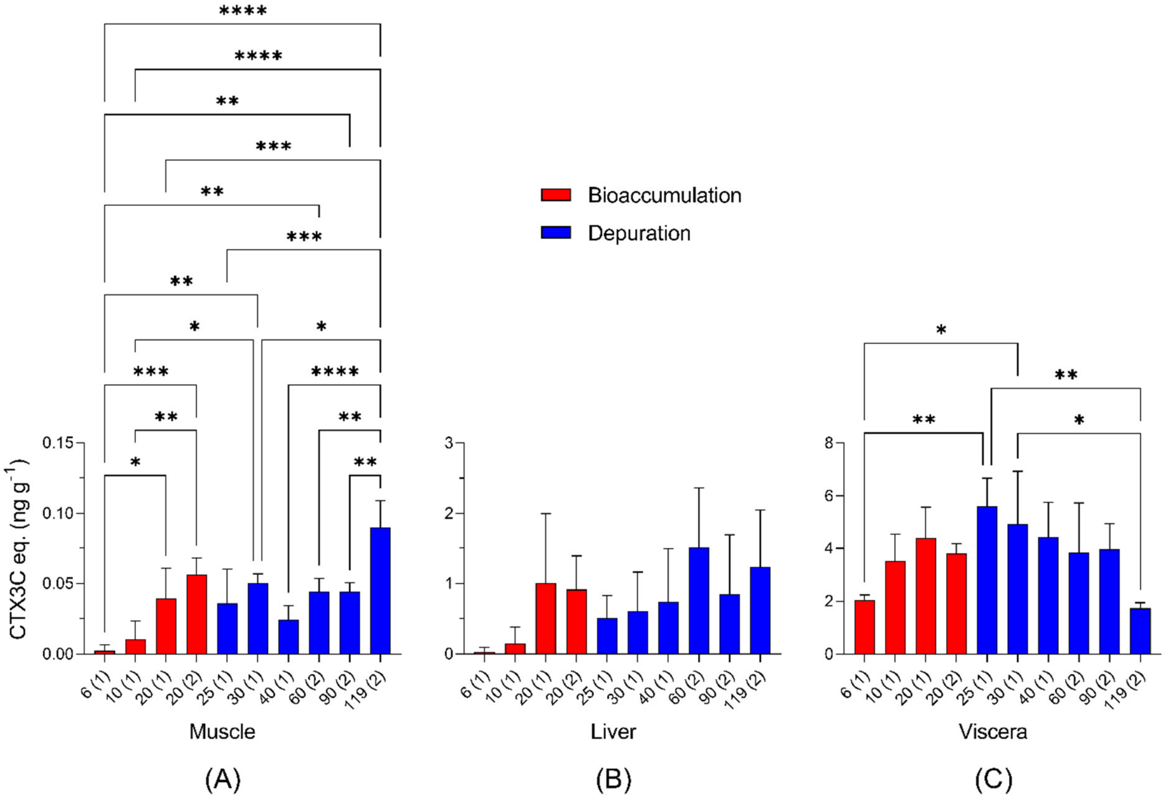

Figure S3. Results of ANOVA with Tukey's test for C-CTX-1 measured in CTX3C eq. concentrations (mean ± s.d.) in (A) muscle, (B) liver, and (C) pooled viscera of L. rhomboides. Fish were fed C-CTX-1 for up to 20 d (red) and depurated for a maximum of 99 d (blue). Numbers along the x-axis indicate the sampling day and trial (in parentheses). Bars represent mean ± s.d. (n = 4; p < *0.05, **0.01, ***0.001, ****0.0001).

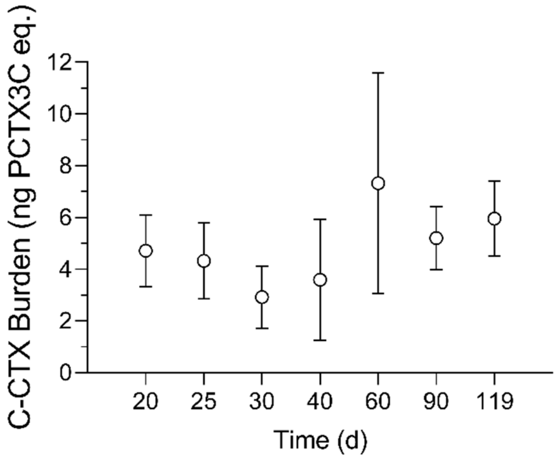

Figure S4. Sum of total C-CTX burdens (measured as CTX3C eq.) in the tissues of *L. rhomboides* sampled in this study. Symbols represent the mean +/- s.d. of n = 4 fish except day 20 (n = 8).
